# Benefit from B-Lymphocyte Depletion Using the Anti-CD20 Antibody Rituximab in Chronic Fatigue Syndrome. A Double-Blind and Placebo-Controlled Study

**DOI:** 10.1371/journal.pone.0026358

**Published:** 2011-10-19

**Authors:** Øystein Fluge, Ove Bruland, Kristin Risa, Anette Storstein, Einar K. Kristoffersen, Dipak Sapkota, Halvor Næss, Olav Dahl, Harald Nyland, Olav Mella

**Affiliations:** 1 Department of Oncology and Medical Physics, Haukeland University Hospital, Bergen, Norway; 2 Department of Medical Genetics and Molecular Medicine, Haukeland University Hospital, Bergen, Norway; 3 Department of Neurology, Haukeland University Hospital, Bergen, Norway; 4 Department of Immunology and Transfusion Medicine, Haukeland University Hospital, and The Gade Institute, University of Bergen, Bergen, Norway; 5 Institute of Internal Medicine, Section of Oncology, University of Bergen, Bergen, Norway; Innsbruck Medical University, Austria

## Abstract

**Background:**

Chronic fatigue syndrome (CFS) is a disease of unknown aetiology. Major CFS symptom relief during cancer chemotherapy in a patient with synchronous CFS and lymphoma spurred a pilot study of B-lymphocyte depletion using the anti-CD20 antibody Rituximab, which demonstrated significant clinical response in three CFS patients.

**Methods and Findings:**

In this double-blind, placebo-controlled phase II study (NCT00848692), 30 CFS patients were randomised to either Rituximab 500 mg/m^2^ or saline, given twice two weeks apart, with follow-up for 12 months. Xenotropic murine leukemia virus-related virus (XMRV) was not detected in any of the patients.

The responses generally affected all CFS symptoms. Major or moderate overall response, defined as lasting improvements in self-reported Fatigue score during follow-up, was seen in 10 out of 15 patients (67%) in the Rituximab group and in two out of 15 patients (13%) in the Placebo group (p = 0.003). Mean response duration within the follow-up period for the 10 responders to Rituximab was 25 weeks (range 8–44). Four Rituximab patients had clinical response durations past the study period. General linear models for repeated measures of Fatigue scores during follow-up showed a significant interaction between time and intervention group (p = 0.018 for self-reported, and p = 0.024 for physician-assessed), with differences between the Rituximab and Placebo groups between 6–10 months after intervention. The primary end-point, defined as effect on self-reported Fatigue score 3 months after intervention, was negative. There were no serious adverse events. Two patients in the Rituximab group with pre-existing psoriasis experienced moderate psoriasis worsening.

**Conclusion:**

The delayed responses starting from 2–7 months after Rituximab treatment, in spite of rapid B-cell depletion, suggests that CFS is an autoimmune disease and may be consistent with the gradual elimination of autoantibodies preceding clinical responses. The present findings will impact future research efforts in CFS.

**Trial registration:**

ClinicalTrials.gov NCT00848692

## Introduction

Chronic fatigue syndrome (CFS)/Myalgic encephalomyelitis (ME) is an illness of unknown aetiology. CFS is characterized by unexplained severe fatigue, excessive post-exercise exhaustion and malaise, sleep disturbances, cognitive impairment, sensory hypersensitivity, muscle and joint pain, headache, bowel symptoms, flu-like episodes and severe impairment of daily functioning. CFS is a major public health problem, with extensive family and public costs for medication, nursing and disability [1], [2]. The prevalence estimates vary up to 1%, but is probably 0.1–0.2% of the population using strict diagnostic criteria [3]. There is at present no established interventional drug treatment for CFS. However, infections frequently precede the outbreak of symptoms and studies point at the immune system being chronically activated [1].

The present line of investigation was initiated after we observed a patient with CFS who experienced unexpected and marked recovery of CFS symptoms lasting for five months during and after cytotoxic chemotherapy for Hodgkin's disease. We recently published the first pilot case series describing clinical activity from B-lymphocyte depletion using the anti-CD20 monoclonal antibody Rituximab in three CFS patients [4]. These observations suggest that B-cells play a significant role in the ongoing clinical features of CFS, and provide clues to possible aetiological mechanisms. To pursue these clues, we performed a randomised, double-blind and placebo-controlled phase II study using Rituximab or saline, in 30 CFS patients.

## Materials and Methods

### Ethics

The study, including amendment, was approved by the Regional Ethical Committee in Norway, no 200800657-9/MRO/400, and by the National Medicines Agency. All patients gave written consent to participate in the study.

### Study design, pre-treatment evaluation, and randomisation

This study (KTS-1-2008, EudraCT no. 2007-007973-22, ClinicalTrials.gov NCT00848692) was a single centre, randomised, double-blind, and placebo-controlled phase II trial comprising 30 patients with chronic fatigue syndrome (CFS). The protocol for this trial and supporting CONSORT checklist are available as supporting information (Protocol S1 and Checklist S1). The main aim was to evaluate the use of B-cell depletion with the anti-CD20 monoclonal antibody Rituximab for CFS symptom relief, during 12 months follow-up. The inclusion criteria were: a diagnosis of CFS by a neurologist, according to the Fukuda 1994 criteria [5], age 18–65 years, and written informed consent. Exclusion criteria were: fatigue not fulfilling CFS criteria, previous malignant disease (except basal cell carcinoma and cervical dysplasia), previous long-term immunosuppressive treatment, previous Rituximab treatment, endogenous depression, lack of ability to adhere to protocol, or evidence of on-going infection.

The primary end-point was effect on CFS symptoms (self-reported and physician-assessed) three months after intervention. The secondary endpoints were effects on CFS symptoms (self-reported and physician-assessed) at 2, 4, 6, 8, 10 and 12 months after intervention, and assessment of toxicity during 12 months follow-up. Overall clinical response was also recorded. In November 2008, an amendment was submitted to the Regional Ethical Committee (approved January 2009), with addition to the initial protocol of secondary endpoints at 8 and 10 months after inclusion. This amendment was based on observation of patient 3 in our pilot case series, who had a major clinical response occurring late after Rituximab intervention [4]. The sample size was estimated based on the initial case series [4], showing response in three out of three consecutive patients, presuming an overall response rate (ORR) of at least 60%, and one or two responders among 15 placebo patients.

**Figure 1:**
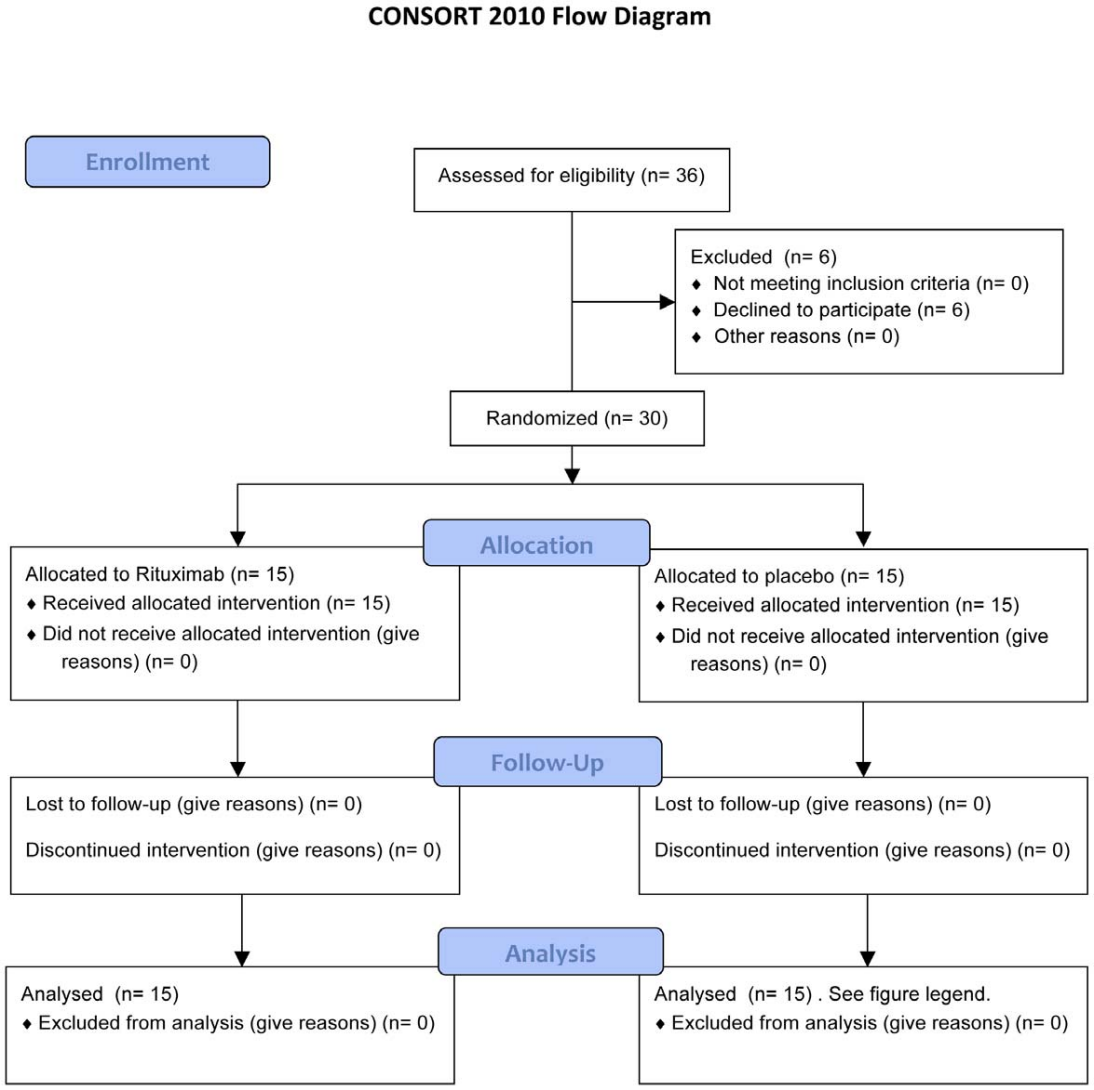
Study flow-diagram. Approximately 60 patients with CFS diagnosed by a neurologist were identified from the hospital files and contacted by telephone for an interview, and 36 of these were invited for a further thorough assessment. During 12 months follow-up, one out of 15 patients in the placebo group was excluded from further analysis after 28 weeks due to pregnancy, and one after 42 weeks due to study withdrawal and patient's decision to start alternative therapy.

Most of the participants were recruited from patients referred to Department of Neurology, Haukeland University Hospital, which is a tertiary referral centre. After examining the patient hospital files from approximately 60 patients, 36 patients who after a telephone interview still seemed to fulfil the inclusion criteria were invited to a new thorough assessment at the Department of Oncology, Haukeland University Hospital (ØF and OM). Six out of these 36 patients chose not to participate (Figure 1).

The pre-treatment evaluation included standard laboratory tests (haematological, liver function, renal function), HCG to exclude pregnancy in fertile women, endocrine assessment (thyroid, adrenal, prolactin), serology for virus (EBV, CMV, HSV, VZV, Enterovirus, Parvovirus B19, adenovirus), immunophenotyping of peripheral blood lymphocyte subsets, common autoantibodies, serum immunoglobulins (IgG, IgM, IgA) with IgG subclasses. MRI of the brain had been performed in all patients. Further diagnostic tests were performed if the pre-treatment evaluation including a thorough clinical examination revealed any relevant abnormality that could explain the severe fatigue experienced by the patients.

The 30 patients were randomly allocated to either treatment with Rituximab or placebo using saline. The randomisation was performed prior to study start by the Pharmacy at Haukeland University Hospital, using a simple (unrestricted) method without blocking. The inclusion period was from June 2008 until June 2009, and the follow-up lasted until June 2010.

According to protocol, four months after the last patient entered the study (end of October, 2009), the patient files and code for intervention, Rituximab versus placebo, were inspected by one of the authors (OD, who did not have patient contact). Based upon his assessment, a decision was made to prolong the blinding until the last included patient had finished eight months follow-up (end of February 2010), at which time 19 patients had finished 10 months follow-up, and 13 patients had finished the complete 12 months observation period. The patients were blinded for group allocation until the end of follow-up (at least 12 months, up to 20 months).

### Treatment schedule, symptom registration, and follow-up

The patients were in-hospital at the Department of Oncology for one night when given the intervention. The infusion bags had double plastic covers to avoid content identification by nurse or patient. Rituximab 500 mg/m^2^ (maximum 1000 mg), diluted in saline to a concentration of 2 mg/ml, or an equal volume of saline, were given twice two weeks apart, with nurse surveillance and according to local guidelines used for treating B-cell lymphomas. No additional Rituximab infusions, or other intervention, were given during follow-up. All patients were given oral cetirizine 10 mg, paracetamol 1 g, and dexamethasone 8 mg prior to infusion.

### CFS symptom scoring

Before intervention, the patients assessed their CFS disease status the last three months and recorded their symptoms according to a visual analogue scale (1–10, 1: no symptom, 10: very severe symptom) (Figure S1). During follow-up, the patients recorded overall change in each symptom the preceding two weeks, as compared to baseline (Figure S2). The visual analogue scale (0–6) for the follow-up scheme was: 0: Major worsening; 1: Moderate worsening; 2: Slight worsening; 3: No change; 4: Slight improvement; 5: Moderate improvement; 6: Major improvement, similar to a Clinical Global Impression Scale used previously in CFS [6]. The patients were assessed at the outpatient clinic pre-treatment, and at 2, 3, 4, 6, 8, 10, and 12 months follow-up. A separate scheme for physician-assessed symptom change was used, with the same scale (0–6) and recorded at all visits (Figure S3). Pre-treatment, and once every month until 10 months follow-up, the patients recorded the SF-36 short form scheme (Norwegian translation), which is a general health-related quality of life questionnaire also assessing eight subdimensions, such as e.g. physical function, bodily pain, social function and mental health. Before treatment, and at 4 months follow-up, the patients were in addition assessed by a neurologist. Patients still in clinical response were followed also after the study period of 12 months.

### Response definitions and statistical analyses

From the self-reported symptoms recorded every second week (Figure S2, scale 0–6), a *Fatigue score* was calculated as the mean of the four symptoms: Fatigue, Post-exertional exhaustion, Need for rest, Daily functioning. The *Pain score* was calculated as the mean of the two pain symptoms assessed to be characteristic for the patient (if pre-treatment level ≥5, among Muscle pain, Joint pain, Headache, Cutaneous pain). The *Cognitive score* was the mean of the three symptoms: Concentration ability, Memory disturbance, Mental tiredness. The *“Other symptoms score”* was derived from the mean of the two symptoms assessed as characteristic for the patient's CFS disease, among those with the highest self-reported pre-treatment level. Also, the patient's self-reported overall interpretation of their CFS disease was recorded *(“CFS overall score”)*. The *Fatigue score, Pain score, Cognitive score, “CFS overall score”*, and *“Other symptoms score”*, were plotted every second week, for each patient in separate diagrams.

The main response was defined from the *Fatigue score*. A major response was defined as a *Fatigue score* ≥4.5 for at least six consecutive weeks, also demanding recordings of some fatigue symptoms as major improvement (value 6) during the response period. A moderate response was recorded as *Fatigue score* ≥4.5 for at least six consecutive weeks, but without recordings of fatigue symptoms as major improvement during the response period. The ORR included both major and moderate responses. The Chi-square test of proportions was used to compare the ORR between the Rituximab and Placebo groups. Improvements in *Fatigue score* with duration less than six weeks were not recorded as significant responses, neither were major improvements in *Cognitive, Pain or “Other symptoms” score* unless followed by a significant improvement in *Fatigue score*.

The self-reported *Fatigue scores* with means for each time interval during follow-up (i.e. 16–24 weeks), and physician-assessed *Fatigue scores*, were plotted. The differences in distribution of *Fatigue scores* for the consecutive time intervals during follow-up, between the Rituximab and Placebo groups, were compared using General Linear Model (GLM) for repeated measures. Separate analyses for self-reported and physician-assessed *Fatigue scores* were made. Five time intervals (with mean *Fatigue scores* in each) were included in the analyses, and Greenhouse-Geisser adjustments were made due to significant Mauchly's tests for sphericity. Main effects for time, for the interaction between time and intervention group, and for the overall difference between groups (Rituximab versus Placebo) were assessed. In addition, the estimates for differences in *Fatigue score* between groups at the five time intervals during follow-up, each level compared to baseline, were generated from the GLM analyses for the interaction time by intervention group (as tests of within-subjects contrasts).

The response durations were defined as the time interval, during 12 months follow-up, with continuous *Fatigue score* ≥4.0 and including the periods of major or moderate responses. The analyses of SF-36 short forms, with physical health summary score, mental health summary score, and scores for the eight SF-36 subdimensions, were generated from a standardised syntax file for each patient before treatment and monthly until 10 months follow-up. The changes in SF-36 scores, compared to baseline, were calculated for each patient during follow-up. For the Rituximab and Placebo groups, the mean baseline SF-36 scores, and the mean values for maximum changes in SF-36 scores during follow-up, were compared using t-tests for independent samples. The statistical analyses were performed using SPSS for Macintosh, version 19.

### Patient characteristics

Between June 2008 and June 2009, 30 patients were randomised. The demographic data and CFS disease characteristics are shown in Table 1. The mean age was 37.3 years (80% female) in the Rituximab group, and 31.5 years (60% female) in the Placebo group. The mean CFS disease duration was 5.1 years in the Rituximab group and 8.1 years in the Placebo group, with maximum disease durations of 13 and 18 years, respectively. A clear or possible clinical infection preceding CFS onset could be identified in 73% and 67% of the patients in the Rituximab and Placebo groups, respectively (Table 1). The frequencies of previous autoimmune diseases for patients and first-degree relatives are also shown (Table 1). The baseline symptom scores were derived from the baseline scheme (Figure S1, scale 1–10) as explained, and are shown in Table 2. The levels of baseline self-reported *Fatigue score*, *Cognitive score*, *Pain score*, *“Other symptoms” score*, and *“CFS overall” score*, were similar in the Rituximab and Placebo groups. Generally, the patients reported a relatively high level of distress for the different symptom scores, while one patient in the Placebo group reported almost no pain (score 1.3), and one patient in the Placebo group reported only mild cognitive symptoms (score 4.0) (Table 2).

**Table 1:** Demographic and CFS disease characteristics for patients in the Rituximab and Placebo groups.

		Rituximab group	Placebo group	p-value^a^
		n = 15	n = 15	
Age, mean (SD)		37.3 years (11.5)	31.5 years (11.6)	0.18
Women, number (%)		12 (80%)	9 (60%)	0.43
CFS disease duration, mean (range)		5.1 years (1.0–13.0)	8.1 years (0.7–18.0)	0.09
Infection before CFS onset	Defined^b^	8	8	0.86
	Possible	3	2	
	No infection	4	5	
Clinical course prior study^c^	Stable	9	12	0.42
	Improvement	1	1	
	Worsening	5	2	
Previous autoimmune disease	In patient^d^	3 (20%)	4 (27%)	1.00
	In first degree relative^e^	5 (33%)	7 (47%)	0.46

a : p-values from student's t-test for continuous data, and from Chi-square tests (or Fisher's exact test) for categorical characteristics.

b : infectious mononucleosis or unspecific viral infection (6), gastroenteritis (4), respiratory infection (5), urinary infection (1).

c : patient's assessment of CFS clinical course the last year before inclusion.

d : thyroiditis (2), psoriasis (2), carpal tunnel syndrome (1), diabetes mellitus type I (1), celiac disease (1), juvenile arthritis (1).

e : Bechterew's disease, rheumatoid arthritis, psoriasis, Cushing's disease, diabetes mellitus type I, systemic lupus erythematosus, thyroiditis, celiac disease, ulcerative colitis, Sjogren's disease, glomerulonephritis.

**Table 2:** Baseline self-reported symptom scores, RNase L genotype, and XMRV status, for patients in the Rituximab and Placebo groups.

		Rituximab group	Placebo group	P-value^a^
		n = 15	n = 15	
Fatigue score^b^, mean (range)		8,1 (7,3–9,8)	7,9 (6,0–9,3)	0.51
Cognitive score		7,7 (5,0–9,7)	7,2 (4,0–9,3)	0.31
Pain score		6,5 (4,0–9,3)	6,2 (1,3–9,0)	0.62
“Other symptoms” score		7,8 (5,5–10,0)	7,9 (5,0–10,0)	0.62
“CFS overall” score		8,3 (7,0–10,0)	7,9 (6,0–10,0)	0.29
Rnase L genotype^c^	462 Q/Q	5	6	
	462 Q/R	10	7	
	462 R/R	0	2	
XMRV status	PCR^d^	0/15	0/15	
	Coculture^e^	0/4	0/5	

a : p-values from Student's t-test.

b : baseline self-reported symptom scores, generated as the mean (range) of relevant symptoms, as explained in Materials and Methods (scale 1–10; 1: no symptom, 5: moderate symptom, 10: very severe symptom, see Figure S1).

c : using Taqman SNP Genotyping (Applied Biosystems, assay c_935391_1).

d : including four Taqman qPCR and four nested PCR setups (see Table S1).

e : performed for nine patients using freshly drawn blood samples. Coculture of patient PBMNC with LNCap prostate cancer cells, and subsequent PCR, as described in Text S1.

### B-lymphocytes

Lymphocyte subpopulations, including CD19 positive B cells, were determined in EDTA anticoagulated blood samples before treatment, and during all follow-up visits (2, 3, 4, 6, 8, 10 and 12 months). Immunophenotyping of lymphocyte subpopulations was performed using the BD Multitest 6-color TBNK kit with BD Trucount Tubes for relative and absolute concentration determination (BD Biosciences). The samples were prepared according to the manufacturers instructions and immediately analysed on a BD Canto II flowcytometer (BD Biosciences). These data were not made available to the clinical researchers before the end of the study.

### Quantitative PCR and PCR for XMRV and MLV, RNase L genotyping

For details of DNA and RNA purification, cDNA synthesis, Quantitative PCR for XMRV detection, XMRV and MLV PCR, Viral amplification, and RNase L genotyping, see Text S1 and Table S1.

## Results

### Clinical responses

Overall response was recorded from the self-reported *Fatigue scores*, defined as lasting improvements for at least six consecutive weeks, independent on when the responses occurred during follow-up. A major response was detected in nine patients in the Rituximab group, and in one patient in the Placebo group (p = 0.002). One patient in the each group achieved a moderate response, giving an total ORR of 10 patients in the Rituximab group (67%, 95%CI 41%–85%) and two patients in the Placebo group (13%, 95%CI 4%–38%), p = 0.003 (Table 3).

**Table 3:** Clinical responses in the Rituximab and Placebo groups, and response durations for patients with significant responses, derived from self-reported *Fatigue scores* during 12 months follow-up.

		Rituximab group	Placebo group	p-value^a^
		n = 15	n = 15	
Clinical responses^b^	Major	9 (60%)	1 (7%)	0.002
	Moderate	1 (7%)	1 (7%)	
	Overall 95% CI	10 (67%) (41%–85%)	2 (13%) (4%–38%)	0.003
Response duration^c^ weeks, mean (range)		25 (8–>44) n = 10	41 (34–>48) n = 2	

a : p-values from Chi-square statistics.

b : One patient was withdrawn from further follow-up registration 28 weeks after inclusion, due to pregnancy. One patient decided to withdraw from the study 42 weeks after inclusion, due to start of alternative therapy. Both patients were allocated to the placebo group and both reported no significant improvement during follow-up. These two were included in the analysis for overall response. Overall response including major and moderate responses, number of patients and proportion (%) with 95% confidence intervals.

c : response duration within the 12 months study period, for those achieving a significant response (10 patients in the Rituximab group, and two patients in the Placebo group). In addition, four out of the 10 Rituximab responders had response durations past the formal study period.

**Figure 2:**
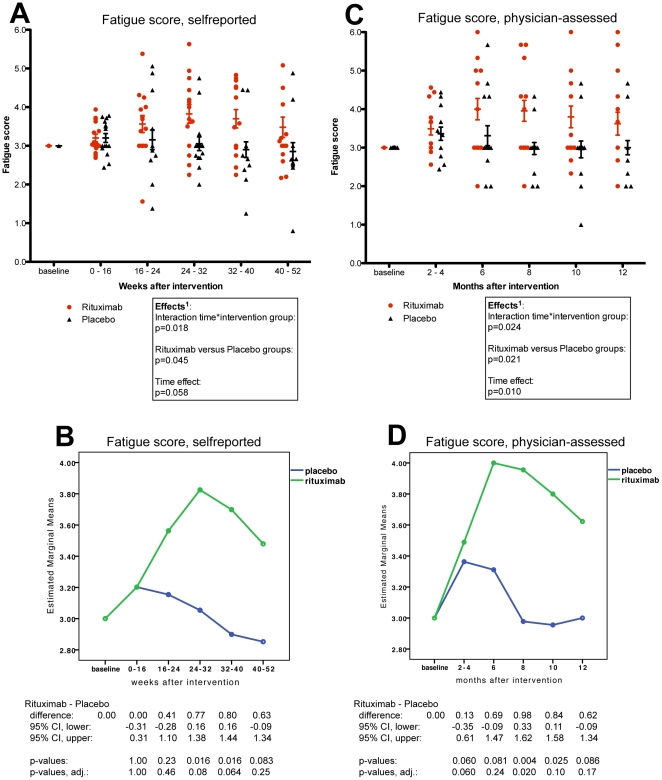
Fatigue scores in Rituximab and Placebo groups, self-reported and physician-assessed. In panel A, the self-reported *Fatigue scores* were calculated for each patient every second week, from the mean of the four symptoms: Fatigue, Post-exertional exhaustion, Need for rest, Daily functioning. Then the mean values in *Fatigue scores* for the time intervals during follow-up were plotted. In panel C, the physician-assessed *Fatigue scores* were calculated from the mean of the same four symptoms, registered by the physician at the visits in the outpatient clinic. In panel B and D, estimated marginal means for self-reported and physician-assessed *Fatigue scores* during follow-up are shown. The scales on Y-axes were 0–6 (0: Major worsening; 1: Moderate worsening; 2: Slight worsening; 3: No change; 4: Slight improvement; 5: Moderate improvement; 6: Major improvement). The differences in distribution of *Fatigue scores* during follow-up, between the Rituximab and Placebo groups, were assessed by General Linear Model (GLM) for repeated measures, analysing the effects of time, the interaction time by intervention group, and the overall difference between intervention groups. Below panels C and D, the estimates for differences in mean *Fatigue scores* between the Rituximab and Placebo groups at the specific time intervals during follow-up, with 95% CI and p-values from the GLM (tests of within-subjects contrasts) are presented. In addition, Holm-Bonferroni step-down adjusted p-values for these time intervals are shown (five comparisons).

The effect of intervention was assessed by studying the interaction effect between time and intervention group, from GLM for repeated measures of self-reported *Fatigue score*. There was a significant interaction time by intervention group in favour of the Rituximab group (p = 0.018), and an overall difference between groups (p = 0.045) (Figure 2, panels A and B, Table S2). Similarly, in GLM for repeated measures of physician-assessed *Fatigue score*, the interaction time by intervention group (p = 0.024) and the overall difference between groups (p = 0.021) were both significant in favour of the Rituximab group (Figure 2, panels C and D, Table S2).

From the GLM tests of within-subjects contrasts for the interaction time by intervention group, with estimates for differences in *Fatigue score* between groups at the specific time intervals, there was no difference in self-reported *Fatigue scores* between the Rituximab and Placebo groups at the predetermined primary endpoint three months after intervention neither from self-reported nor from physician-assessed symptom scores (Figure 2, panels A–D). The differences in *Fatigue scores* between intervention groups were most evident between 6–10 months after intervention (Figure 2, panels A–D), i.e. corresponding to secondary endpoints of the study.

The response durations within 12 months follow-up are shown in Table 3, with mean duration 25 weeks (range 8–44) for the 10 responders in the Rituximab group. Interestingly, four patients in the Rituximab group, and one patient in the Placebo group, had response durations beyond the study period of 12 months, i.e. still *Fatigue score* ≥4 at 12 months. At the time of submission of this manuscript, two patients in the Rituximab group, and one patient in the Placebo group, have lasting major responses for all CFS-related symptoms without signs of relapse, 32, 30, and 23 months after intervention, respectively.

**Figure 3:**
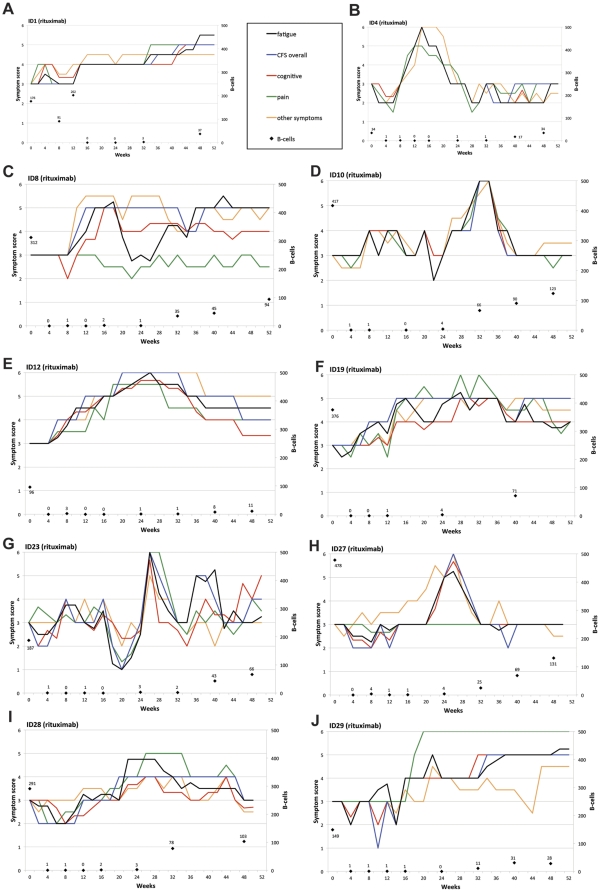
CFS symptom changes during follow-up for patients in the Rituximab group with significant responses. In panels A–J, changes in *Fatigue score* (black), *Cognitive score* (red), *Pain score* (green), *“Other symptoms” score* (orange), and *“CFS overall” score* (blue), during 12 months follow-up are shown for the 10 patients in the Rituximab group with significant improvement. The scales on Y-axes were 0–6 (0: Major worsening; 1: Moderate worsening; 2: Slight worsening; 3: No change; 4: Slight improvement; 5: Moderate improvement; 6: Major improvement). Also shown are the B-cell numbers from immunophenotyping of peripheral blood mononuclear cells during follow-up (×10^6^/L).

The self-reported symptoms scores were plotted every second week for each patient. These data are shown for the 10 responders in the Rituximab group (Figure 3, panels A–J), and for the two patients with significant improvement in the Placebo group (Figure S4, panels A–B). These charts show that symptoms in all the four main categories tend to follow each other in time. Also included in these plots are the B-cell numbers during follow-up.

The SF-36 short form, registering health related quality of life, was filled-in by the patients at baseline and every four weeks during follow-up. Two patients in the Rituximab group did not fill in baseline SF-36 form and were excluded from these analyses, one with a major response and one with no response during follow-up. The patients reported a low baseline “physical health summary score”, with a mean value of 24 in the Rituximab and 26 in the Placebo group (scale 0–100, lower value denotes worse symptoms), while the baseline “mental health summary score” was higher with a mean value of 46 in both groups (Table 4). There were no differences in baseline levels for the different SF-36 subdimensions, except for “general health”, which was lower in the Rituximab group than in the Placebo group (Table 4). During follow-up, the maximum changes in SF-36 scores (as compared to the baseline level) were significantly different in favour of the Rituximab group, for “physical health summary score” (p = 0.039), and the subdimensions “physical function” (p = 0.014) and “bodily pain” (p = 0.005), while there were trends for difference for “general health” (p = 0.081) and “social function” (p = 0.081). There were no differences in maximum changes (compared to baseline) during follow-up between the Rituximab and Placebo groups, for instance for the subdimensions “role emotional” (p = 0.44) or “mental health” (p = 0.81) (Table 4).

**Table 4:** SF-36 scores, baseline levels (0–100) and maximum changes (%) during follow-up, for patients in the Rituximab and Placebo groups.

		Rituximab group	Placebo group	p-value^a^
		n = 13^b^	n = 15	
Physical health summary score	baseline^c^, mean (SD)	24 (5)	26 (6)	0.41
	max change^d^, mean (SD)	54% (46)	26% (17)	0.039
Mental health summary score	baseline, mean (SD)	46 (11)	46 (8)	0.99
	max change, mean (SD)	9% (54)	5% (32)	0.84
Physical function	baseline, mean (SD)	34 (6)	35 (7)	0.71
	max change, mean (SD)	39% (33)	11% (22)	0.014
Role physical	baseline, mean (SD)	28 (8)	28 (2)	0.92
	max change, mean (SD)	27% (35)	20% (35)	0.64
Bodily pain	baseline, mean (SD)	32 (8)	34 (9)	0.62
	max change, mean (SD)	40% (31)	8% (24)	0.005
General health	baseline, mean (SD)	27 (5)	33 (6)	0.007
	max change, mean (SD)	36% (51)	9% (23)	0.081
Vitality	baseline, mean (SD)	35 (8)	31 (5)	0.15
	max change, mean (SD)	24% (50)	31% (30)	0.66
Social function	baseline, mean (SD)	21 (9)	21 (8)	0.92
	max change, mean (SD)	98% (110)	38% (62)	0.081
Role emotional	baseline, mean (SD)	50 (12)	51 (11)	0.74
	max change, mean (SD)	10% (53)	−4% (42)	0.44
Mental health	baseline, mean (SD)	49 (9)	50 (7)	0.69
	max change, mean (SD)	6% (36)	3% (22)	0.81

a : p-values from Student's t-test for independent samples.

b : two patients in the Rituximab group did not fill in baseline SF-36 form, one with major response and one non-responder.

c : range for baseline SF-36 scores is 0–100 (lower score denotes increasing symptoms).

d : maximum change (%) in SF-36 scores during follow-up, as compared to baseline. The patients filled in SF-36 forms at baseline and then every month until 10 months after intervention. Five out of 143 forms were missing (not filled in) in the Rituximab group (excluding the two patients with no baseline scheme), and nine out of 161 forms were missing in the Placebo group (one patient withdrawn after 28 weeks due to pregnancy).

In spite of written and verbal information to avoid pregnancy, one patient was pregnant after 28 weeks follow-up. The randomisation code for this patient was then revealed. She was allocated to the placebo group and was withdrawn from further follow-up registration after 28 weeks. One patient decided to withdraw from the study after 42 weeks follow-up to start alternative therapy. This patient proved also to be allocated to the placebo group.

### Adverse effects

Infusion-related complaints and side-effects during 12 months follow-up are listed in Table 5. There were no serious adverse events or major toxicity. The complaints during infusion or the following day were mild and also reported by the placebo patients (five in the Rituximab group and four in the Placebo group). Two patients in each group reported slight worsening of CFS the first two months after intervention (Table 5). Two patients in the Rituximab group reported feeling uneasy and sleepless from 2–7 and 6–8 months, respectively, synchronous with a clear clinical response. Two patients in the Rituximab group with pre-existing psoriasis experienced moderate psoriasis worsening from two months after intervention, in both preceding a clinical response of CFS. One patient in the Rituximab group had a five years history of major CFS symptoms, and reported some lower back pain and episodes of balanitis from 5–7 months, synchronous with improvement of his CFS-related muscle pain and headache and with improving fatigue and cognitive function. His low back pain disappeared, and after the 12 month study period his CFS symptoms have resolved further and he is at present back in full-time work 30 months after intervention.

**Table 5:** Infusion-related complaints during or the first 24 hours after infusion, and side-effects during 12 months follow-up, in the Rituximab and Placebo groups.

		Rituximab group	Placebo group
		n = 15	n = 15
Infusion-related complaints	Palpitations	1 (7%)	1 (7%)
	Slight itching	2 (13%)	0
	Nausea	0	1
	Discomfort	2 (13%)	2 (13%)
Slight CFS worsening the first two months		2 (13%)	2 (13%)
Irregular menstrual bleeding the first two months		1 (7%)	0
Feeling uneasy and sleepless	6–8 months	1 (7%)	0
	2–7 months	1 (7%)	0
Slight facial acne		1 (7%)	0
Psoriasis worsening	2–12 months	2 (13%)	0
Low back pain and balanitis	5–7 months	1 (7%)	0

There were some uncomplicated upper airways infections and lower urinary tract infections during follow-up, evenly distributed between the two groups. There were two hospitalisations during the study period within 12 months follow-up, one in the Rituximab group due to abdominal pain where a laparoscopy showed a corpus luteum cyst interpreted as a normal finding, and one in the Placebo group due to a myocardial infarction.

### XMRV

In this study, XMRV could not be detected in any of the 30 patients, using four Taqman quantitative PCR setups, and four nested PCR approaches (Text S1 and Table S1), and using both genomic DNA and cDNA from baseline blood samples as templates. When performing coculture of patient lymphocytes with LNCaP prostate cancer cells for biological amplification prior to PCR, XMRV was not detected in any sample. The positive control was readily amplified in all setups, and both sensitivity and specificity of the Taqman assays proved to be high, detecting 5–15 viral copies per vial with dilution series of the plasmid spiked in normal genomic DNA background (data not shown). For details on analyses, see Supporting information.

### RNase L

Results from genotyping of the RNase L Q462R variant are shown in Table 2. Two patients in the Placebo group had the 462 R/R genotype. The distribution of the Q/Q, Q/R, and R/R alleles was close to that expected in the general population.

### B-lymphocytes

**Figure 4:**
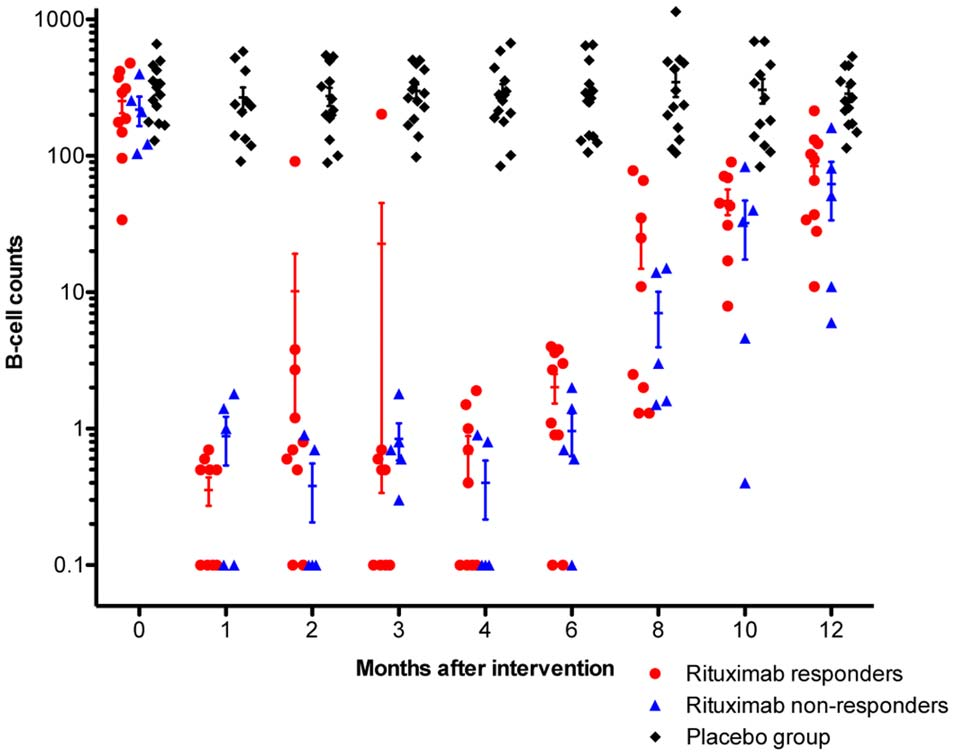
B-lymphocytes during follow-up. B-cell numbers from immunophenotyping of peripheral blood during follow-up are shown, for patients in the Placebo group (black, n = 15), patients in the Rituximab group with significant response (red, n = 10), and patients in the Rituximab group with no response (blue, n = 5). The B-cell value zero was substituted by 0.1 (to be able to plot on the log scale). B-lymphocyte counts ×10^6^/L (normal range 110–449). The error bars denote mean ± SEM.

Results from immunophenotyping with B-cell counts, for patients in the Rituximab group (responders and non-responders) and the Placebo group, from baseline and at all visits at 1, 2, 3, 4, 6, 8, 10, and 12 months, are shown in Figure 4. At baseline, three out of 30 patients had a B-cell count below the normal range (110–449×10^6^/L). All patients in the Rituximab group were B-cell depleted at one month after the intervention, except for one patient in which B-cells were not adequately depleted until four months after intervention (Figure 4). During follow-up, there were no differences in B-cell levels between patients in the Rituximab group achieving a response (n = 10), and those with no significant response (n = 5) (Figure 4). Many patients treated with Rituximab were not completely B-cell depleted, with values in the range 0–4 (×10^6^/L) (Figure 4). After two infusions of Rituximab two weeks apart, the B-cell levels started to increase from 4–8 months after intervention, and four out of 15 patients had regained B-cell counts in the normal range at the end of follow-up at 12 months. We also performed Taqman qPCR for the B-cell specific CD19 molecule, normalised according to β-actin mRNA, at baseline and during follow-up at 4, 6 and 8 months. These data confirmed the B-cell depletion in Rituximab-treated patients but could not separate the Rituximab responders and non-responders (data not shown).

## Discussion

In this double-blind and placebo-controlled study, treatment with Rituximab (two infusions two weeks apart) was associated with a significant overall clinical response during 12 months follow-up, as compared to placebo using saline. The ORRs were 67% in the Rituximab group and 13% in the Placebo group.

From GLM for repeated measures, there were significant interactions between time and intervention group in favour of the Rituximab group both for self-reported and physician-assessed *Fatigue scores*. The differences were most evident corresponding to secondary endpoints between 6 and 10 months after intervention. However, at the predetermined primary endpoint, defined as effect on CFS symptoms (*Fatigue score*) at three months after intervention, there was no difference between the groups.

The change in *Fatigue scores* was chosen as the main criterion for clinical response because this is a key symptom defining the CFS disease. However, with few exceptions, the different symptoms scores (*Fatigue, Cognitive, Pain, “Other symptoms”, “CFS overall”*) followed each other during responses and relapses, indicating that a central mechanism for the symptom maintenance is influenced by the B-cell depletion treatment.

For the 10 Rituximab responders, the response durations varied from 8–44 weeks (within the 12 month study period). These responses were considered to have a significant impact on symptom improvement and quality of life by the patients, by their families, and by the treating physicians. Importantly, four patients in the Rituximab group and one in the Placebo group had response durations beyond 12 months. When submitting this manuscript, two patients in the Rituximab group and one in the Placebo group have lasting major responses with no signs of relapse and are back in full-time work. Two of these patients (one in each group) had the shortest disease durations, 1.0 and 0.7 years, respectively, and in both CFS was preceded by Epstein-Barr infection. These two lasting responses may be examples of spontaneous recovery [7]. The third “long-time responder” with no later relapse had a 5-year history with major CFS symptoms before entering the study.

This study with the primary endpoint at three months after intervention was designed when we had very limited experience with B-cell depletion in CFS, having observed only two pilot patients, both with an early response pattern. In the pilot case series [4] the third patient had major response occurring from 5 ½ until 9 months after intervention, and with an almost identical response and relapse pattern after her second Rituximab treatment. In the present study, only one out of 10 responders in the Rituximab group had an “early” response pattern with the major response starting from 2 months, while the remaining nine responded later, starting to improve from between 3–7 months after treatment.

The SF-36 short form analyses before intervention confirmed a low quality of life particularly for the dimensions related to physical health, while the subdimensions “mental health” and “role emotional” showed higher baseline values. In support for the overall clinical response data, SF-36 analyses during follow-up demonstrated significant differences in favour of the Rituximab group in maximum changes compared to baseline level, for several subdimensions related to physical health, such as “physical health summary score”, “physical function” and “bodily pain”. There were no significant differences between the groups in maximum changes for in the subdimensions “mental health” or “role emotional”. These results support the assumption that CFS is not primarily a mental health disease.

The lack of specific markers of disease and the fact that the CFS diagnosis is controversial imply that the level of diagnostic accuracy and adherence to international criteria are likely to be variable. In this study, patients have been recruited from a tertiary referral centre and were re-evaluated by the researchers, using the Fukuda criteria for inclusion. Retrospectively, we checked the 30 patients according to the Clinical Working Case Definition (“Canadian criteria”) [8]. Two patients in the placebo group did not fulfil these criteria, and it could therefore be argued that they might not have CFS. One of these had almost no pain, and one had only slight cognitive symptoms and also reported marked mood disturbances. The latter patient reported major improvement after intervention with saline and was recorded as one of the two responders in the Placebo group. Generally, the patients reported a high level of disease-related symptoms prior to entering the study, with major impact on daily life and ability to participate in family and social activities. Most of the patients had a long CFS disease duration, also with a stable or worsening clinical course the last year before study entry.

A limitation of this study is the lack of predetermined exact definitions of clinical response with respect to duration and extent of improvement. Limited experience with the clinical course of CFS after B-cell depletion when designing the protocol, with erroneous estimation of time frame for the clinical responses, is a main reason why the primary end-point is negative. Therefore, even though this is a randomised, double-blind and placebo-controlled study, there are also exploratory elements with respect to response characterization.

Even when interpreting the results with caution, there are obvious differences between the two groups in favour of the Rituximab group, from the significant interactions time by intervention group and also from difference in ORR. Thus, we have shown that in a subset of CFS patients, Rituximab treatment with subsequent B-cell depletion results in major responses of CFS related symptoms, with responses starting to occur between 2 and 7 months after intervention, and with response durations of 2–15 months and in addition two patients with no relapse. Five patients in the Rituximab group did not achieve a significant response during follow-up. Whether their CFS pathogeneses are unrelated to B-lymphocytes, or whether they could achieve a clinical response through prolonged B-cell depletion with Rituximab maintenance therapy is at present unknown.

The associations between B-cell depletion and clinical responses, and the time frames for clinical responses delayed 2–7 months after the initial and rapid B-cell depletion, indicate that CFS may be an autoimmune disease, often preceded by an infection, and targeting specific parts of the nervous system. We speculate that the responses occurring late after intervention could be explained by the elimination of disease-associated autoantibodies, while the early response pattern could be related to interaction of B-cells with T-cells in antigen presentation, as suggested in systemic lupus erythematosus [9]. Work is in progress in our laboratory to elucidate the localization and nature of a putative target for an autoimmune process.

The high rate of CFS in women compared to men is a suggestion of an underlying autoimmune process. On-going autoimmune phenomena in CFS have been discussed [10] perhaps triggered by infections through molecular mimicry, through structural similarity between a pathogen component and self-structures [11]. Several autoantibodies have been reported in CFS, but their pathogenic roles have not been established, for a review see [10]. In the present study, 23% of the patients had a previous known autoimmune disease, and 40% had first-degree relatives with an autoimmune disease. However, there were low frequencies of positive known common autoantibodies, none had elevated anti-nuclear antibodies, and two had elevated anti-thyroid peroxidase antibodies (data not shown). A possible or established clinical infection before CFS onset was identified in 21 out of 30 patients included (70%).

A recent study of cytokine patterns in CFS showed attenuated Th1 and Th17 immune responses, while there was high Th2 marker expression [12]. A Th2 profile with increase in the numbers of CD4+ and CD8+ T cells secreting IL-4 following polyclonal stimulation, including in resting cells, was demonstrated in CFS patients [13].

There are limited data on the role of B-lymphocytes in CFS. One study of gene expression in peripheral blood mononuclear cells indicated that B cells might be altered in CFS [14]. Our pilot case series [4] suggested that B-cell depletion was associated with clinical improvement in CFS patients. B-cell targeting therapy has been increasingly used for diverse autoimmune diseases in later years [15]. The monoclonal anti-CD20 antibody Rituximab has been the major B-cell depleting agent so far. The B-cells have multiple immune functions, the main ones being antibody production, antigen presentation and regulation of the function and activity of other immune cells, i.e. T-regulatory cells, NK cells and macrophages [16]. Thus, the total effects of B-cell depletion on the immune system are likely to be complex and time-dependent.

In Stiff Person Syndrome, which is a rare autoimmune disease in the central nervous system with autoantibodies targeting glutamic acid decarboxylase (GAD65), the humoral autoimmune response was shown to consist of a Rituximab-sensitive part rapidly cleared after treatment, and a Rituximab-resistant part from long-lived and persistent plasma cells acting as a reservoir for secretion of autoantibodies. This may be one mechanism for lack of effect from B-cell depletion therapy [17].

An alternative explanation for the observed clinical improvement from B-cell depletion could be elimination or reduction of B-lymphotrophic viruses such as cytomegalovirus (CMV) or Epstein-Barr virus (EBV). A recent study [18] showed clinical benefit from long-term valacyclovir or valgancicliovir treatment in a subset of CFS patients with evidence of active on-going herpes virus infections, also shown in a previous small study of 12 patients with elevated antibodies to EBV or human herpes virus 6 (HHV-6) [19]. We find the delayed clinical responses after B-cell depletion, that we observed in the majority of responders, difficult to explain from a viral elimination mechanism. In our opinion, this response pattern is more compatible with the gradual elimination of an autoantibody, perhaps by preferential elimination of short-lived pre-plasma cells. Although poorly understood, immune system alterations seen in stress-related diseases such as post-traumatic stress disorder could also be relevant for the effects of B-cell depletion seen in CFS [20].

In this study there were no serious adverse events. Two hospitalisations (one in each group) during follow-up were not perceived to be caused by the intervention. There were no serious infections. Two patients in the Rituximab group (both responders) had a slight to moderate worsening of pre-existing psoriasis, which could be a direct side-effect from the intervention. Two patients in the Rituximab group (also responders) felt uneasy and sleepless while improving in CFS symptoms. These symptoms are not usual side effects from treatment of B-cell lymphomas and may be related to the effects of B-cell depletion on the underlying CFS disease rather than direct side-effects from Rituximab. The infusion-related complaints were generally mild and reported both from the Rituximab and Placebo groups. Interestingly, no side-effects during follow-up were reported from the five non-responders in the Rituximab group.

Current use of Rituximab in autoimmune diseases has been discussed [21], and a recent review on the safety in rheumatoid arthritis concluded that Rituximab is well tolerated with no increasing rate of serious infections [22]. Even if serious side-effects including reactivation of viral infections such as hepatitis B are very rare, they may appear [23]. A study reviewing progressive multifocal leukoencephalopathy (PML) cases in patients treated with Rituximab, from available databases including the Food and Drug Administration, the manufacturer, physicians, and including a literature review from 1997–2008, identified 52 patients with lymphoproliferative disorders and five patients with autoimmune diseases [24]. All had received either chemotherapy or other immunosuppressive treatment in addition to Rituximab, and to our knowledge PML has not been reported in patients receiving Rituximab monotherapy. The absolute risk of PML is difficult to estimate, but is very low.

Although B-cell depletion has a major impact on the immune system, it can be argued that the persistent and often devastating symptoms of CFS and the very low quality of life in many sufferers justify the use of Rituximab in carefully evaluated CFS patients.

There has been an intense focus on XMRV as a possible causal gammaretroviral infection in CFS patients since the first report in October 2009 [25]. One later study demonstrated presence of closely related MLV in CFS patients [26]. However, several other studies have not been able to reproduce these findings and reported no evidence for XMRV infection in CFS, as examples [27]–[30]. We have not been able to demonstrate XMRV or MLV infection in any of the 30 patients included in this study, despite multiple different approaches including four different highly sensitive Taqman qPCR and four nested PCR approaches, and using both genomic DNA and cDNA as templates. We also performed coculture of patient peripheral blood mononuclear cells with LNCaP prostate cancer cells for biological virus amplification prior to PCR, also with no XMRV positive cases.

In conclusion, we have shown that B-lymphocyte depletion with the monoclonal anti-CD20 antibody Rituximab, two infusions two weeks apart, is associated with significant, though generally transient clinical responses, with CFS symptom improvement in two thirds of the included patients. No major toxicity was observed in 12 months follow-up. Even though this study is small, it is a randomised, double-blind, and placebo-controlled study showing significant differences in favour of the Rituximab group, and with general improvement of all CFS symptoms. Thus, we believe that B-cell depletion targets a central player in the pathogenesis of the CFS disease, directly or indirectly. Whether this mechanism applies to specific subsets of CFS patients or the group as a whole is a subject for further research. While this study can be interpreted as preliminary, being the first to describe the treatment principle in CFS except for our pilot case series [4], we believe the results are best compatible with an autoimmune disease mechanism and that the presented findings may have a major impact on the direction of biomedical research in CFS. Based on new pilot patient experiences, we have now started two new open-label phase-II studies investigating Rituximab treatment with two infusions two weeks apart (as in the present study) followed by maintenance Rituximab infusions at 3, 6, 10 and 15 months, to further explore this treatment principle in CFS (ClinicalTrials.gov, NCT01156909 and NCT01156922). The present study may be interpreted as a “proof of principle”. The next studies will indicate to what extent the patients actually over time may recover through B-cell depletion treatment.

## Supporting Information

Text S1 **Materials and Methods** **(DNA and RNA purification, and cDNA synthesis.** Quantitative PCR for XMRV detection. XMRV and MLV PCR. Viral amplification. RNase L Genotyping).
(PDF)

Figure S1 **Scheme for patient's self-reported baseline CFS symptoms.** Before intervention, the patients assessed their CFS disease and recorded their symptoms the last three months according to a visual analogue, scale 1–10 (1: no symptom; 5: moderate symptom; 10: very severe symptom).
(TIF)

Figure S2 **Scheme for patient's self-reported CFS symptom change during follow-up.** During 12 months follow-up, the patients recorded symptom changes the preceding two weeks, as compared to baseline. The visual analogue scale for the follow-up scheme was 0–6 (0: Major worsening; 1: Moderate worsening; 2: Slight worsening; 3: No change; 4: Slight improvement; 5: Moderate improvement; 6: Major improvement).
(TIF)

Figure S3 **Scheme for physician-assessed CFS symptoms, at baseline and during follow-up.** The patients were assessed at the outpatient clinic before intervention, and at 2, 3, 4, 6, 8, 10, and 12 months follow-up. The physicians assessed the patients CFS disease and recorded the symptoms according to visual analogue scales. Before intervention, the scale was 1–10 (1: no symptom; 5: moderate symptom; 10: very severe symptom). During 12 months follow-up, the physicians assessed patients symptom changes as compared to baseline, scale 0–6 (0: Major worsening; 1: Moderate worsening; 2: Slight worsening; 3: No change; 4: Slight improvement; 5: Moderate improvement; 6: Major improvement).
(TIF)

Figure S4 **CFS symptom changes during follow-up, for the two patients in the Placebo group with significant improvement.** In panels A and B, changes in *Fatigue score* (black), *Cognitive score* (red), *Pain score* (green), *“Other symptoms” score* (orange), and *“CFS overall” score* (blue), during 12 months follow-up are shown for the two patients in the Placebo group with significant improvement. The scales on Y-axes were 0–6 (0: Major worsening; 1: Moderate worsening; 2: Slight worsening; 3: No change; 4: Slight improvement; 5: Moderate improvement; 6: Major improvement). Also shown are the B-cell numbers from immunophenotyping of peripheral blood mononuclear cells during follow-up (×10^6^/L).
(TIF)

Table S1 **Primers and probes for detection of Xenotropic murine leukemia virus-related virus (XMRV) and MLV-related virus.**
(PDF)

Table S2 **Effects of intervention group (Rituximab versus Placebo) on** ***Fatigue score*** **during 12 months follow-up, using General Linear Model for repeated measures, with separate analyses for self-reported and physician-assessed symptoms.**
(DOC)

Protocol S1 **Trial Protocol.**
(PDF)

Checklist S1 **CONSORT Checklist.**
(PDF)
