# Supplementary material for: Benefit from B-Lymphocyte Depletion Using the Anti-CD20 Antibody Rituximab in Chronic Fatigue Syndrome. A Double-Blind and Placebo-Controlled Study
Source: PLoS One. 2011 Oct 19;6(10):e26358. doi: 10.1371/journal.pone.0026358 (PMC3198463; doi:10.1371/journal.pone.0026358)
Supplement: Text S1 — Materials and Methods (DNA and RNA purification, and cDNA synthesis. Quantitative PCR for XMRV detection. XMRV and MLV PCR. Viral amplification. RNase L Genotyping). (PDF) [file pone.0026358.s001.pdf]

Supporting information. Text S1

Materials and methods

DNA and RNA purification, and cDNA synthesis

From all patients and controls, before intervention and from all follow-up visits (at 2, 3, 4, 6, 8, 10, 12 months) one vial of EDTA blood, three vials of heparin blood, three vials of serum, one vial of plasma, and two Tempus blood RNA tubes (Applied Biosystems, cat.no.4342792) were collected and frozen at -80^{0}C.
DNA was purified from 200 µl EDTA blood using the QIAamp DNA Blood Mini Kit (Qiagen, cat # 51106). DNA was purified from confluent LNCaP cells using the Blood and Cell culture DNA Mini Kit (Qiagen, cat # 13323). RNA was purified from Tempus blood RNA tubes using the Tempus Spin RNA Isolation kit (Applied Biosystems, P/N 4380204). Total RNA was isolated from confluent LNCaP/PBMC cells using the Qiagen RNeasy Mini kit (Qiagen, cat # 74104). Purity and concentration of DNA and of RNA were determined by UV-spectroscopy using a NanoDrop 1000 Spectrophotometer (Thermo Scientific Inc).
cDNA was synthesized from total RNA using the High capacity cDNA reverse transcription kit (Applied Biosystems, P/N 4375222). 200 ng total RNA was used as template in a total volume of 20 µl, following the manufacturers instructions.

Quantitative PCR for XMRV detection

Quantitative RT-PCR was performed on the ABI 7900 instruments (Applied Biosystems). Each real time PCR reaction contained either 0.5 µl cDNA or 1 µl gDNA, 4 µl Taqman Universal Master mix (Applied Biosystems), 420 nM sense and anti-sense primer, 93.5 nM TaqMan probe in a total volume of 8 µl. Each sample was run in triplicate. Cycling parameters were 95ºC for 10 min, followed by 40-50 cycles of 95ºC for 15 s and 60ºC for 1 min. Taqman β-actin detection reagent (Applied Biosystems, cat # 4310881E) was used as endogenous normalisation control to adjust for unequal amounts of RNA, while pmp22 were used as reference control when gDNA was used as template (Supplementary data, Table 1).
For detection of XMRV from peripheral blood mononuclear cells, four different Taqman setups were performed (Supplementary data, Table 1), using templates from pre-treatment samples of all 30 patients in the study, and performed both using genomic DNA and cDNA as input. For Taqman qPCR experiments, the template amounts varied from 20 to 500 ng for RNA/cDNA, and from 200 to 500 ng for genomic DNA. The positive control (VP62 plasmid, a gift from R. Silverman) was readily amplified from all qPCR setups.

XMRV and MLV PCR

Single round PCR screening for the presence of XMRV was performed with two sets of primers, one (XMRV_5922F and XMRV_6273R primers) amplifying a region located in the env coding region of the virus, and one (gag 419F and gag 1154R primers) amplifying from the gag coding region (Supplementary data, Table 1). The PCR conditions were 600 nM of each primer, 2 mM $MgCl_2$, 2.5 U AmpliTaq Gold (Applied Biosystems), 0.8 mM dNTP, 1 x reaction buffer in a total volume of 25 µl. PCR cycling were 94ºC for 10 min, then 45 cycles of 94ºC for 30 sec, 57ºC for 30 sec and 72 ºC for 1 min, followed by an extension step at 72ºC for 7 min.

Nested PCR for detection of XMRV or MLV was performed essentially as described[1,2], but using four different setups, as shown in Supplementary data, Table 1. Both the first PCR rounds and then the second rounds were performed for 40 cycles. Ordinary PCR and nested PCR experiments were performed from pre-treatment samples of all 30 patients. In two setups both genomic DNA and cDNA were used as templates, and in two setups only genomic DNA templates were used (Supplementary data, Table 1). The VP62 plasmid was used as positive control for all XMRV PCR setups.

Viral amplification

Viral amplification was performed using fresh blood samples from nine patients included in the study, as previously described[2] (see also: http://www.iacfsme.org/BULLETINSPRING2010/Spring2010MikovitsLetter/tabid/427/Default.aspx). Briefly, PBMC were isolated from heparin peripheral blood by Ficoll centrifugation and incubated for three days in T-25 tissue culture flasks (Nunc) containing RPMI-1640 medium supplemented with 10 % fetal bovine serum, 2 mM glutamine, 1 mM sodium pyruvate, antibiotics (PenStrep), phytohemagglutinin (1 µg/ml) and IL-2 (20 U/ml). Following activation, 1 x 10^5 PBMC free of IL-2 were mixed with 5 x 10^5 detached LNCaP cells in 250 µl complete RPMI medium and 250 µl autologous plasma, and centrifuged at 1500 rpm for 10 min. Cell pellets were dissolved in complete RPMI medium (10 % fetal bovine serum, 2 mM glutamine, 1 mM sodium pyruvate) in T-25 flask and incubated until confluence. The cells were harvested, and genomic DNA and RNA purified, with cDNA synthesis as described above. Four Taqman PCR setups, and one nested PCR, were performed from both genomic DNA and cDNA as templates (Supplementary data, Table 1).

RNase L Genotyping

Genotyping of the RNase L Q462R variant, by investigating dbSNP ID rs486907, was performed on the ABI PRISM 7900 Sequence Detection System using Taqman SNP Genotyping (Applied Biosystems, assay c_935391_1) according to the manufacturers recommendations. Briefly, each well included 2.5 µl 2 x PCR master mix (Applied Biosystems), 0.25 µl SNP Genotyping Assay Mix, and approximately 20 ng genomic DNA in a total volume of 5 µl. All samples were run in triplicates.

References

1. Lo SC, Pripuzova N, Li B, et al. Detection of MLV-related virus gene sequences in blood of patients with chronic fatigue syndrome and healthy blood donors. *Proc Natl Acad Sci U S A* 2010; 107: 15874-9.
2. Lombardi VC, Ruscetti FW, Das Gupta J, et al. Detection of an Infectious Retrovirus, XMRV, in Blood Cells of Patients with Chronic Fatigue Syndrome. *Science* 2009; 326: 585-9.
