# Supplementary figures and images for: Benefit from B-Lymphocyte Depletion Using the Anti-CD20 Antibody Rituximab in Chronic Fatigue Syndrome. A Double-Blind and Placebo-Controlled Study
Source: PLoS One. 2011 Oct 19;6(10):e26358. doi: 10.1371/journal.pone.0026358 (PMC3198463; doi:10.1371/journal.pone.0026358)

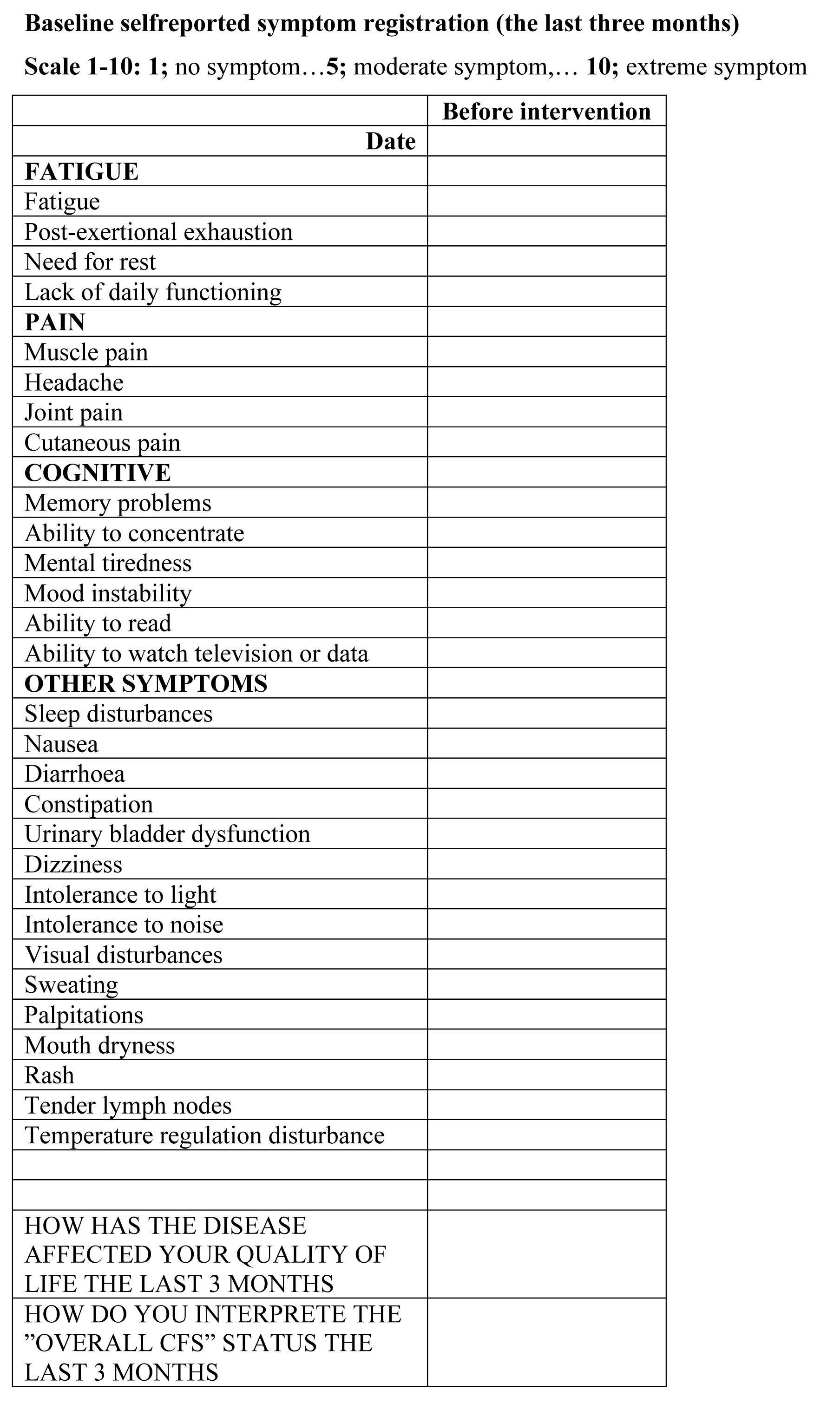

Supplement: Figure S1 — Scheme for patient's self-reported baseline CFS symptoms. Before intervention, the patients assessed their CFS disease and recorded their symptoms the last three months according to a visual analogue, scale 1–10 (1: no symptom; 5: moderate symptom; 10: very severe symptom). (TIF) [file pone.0026358.s002.tif]

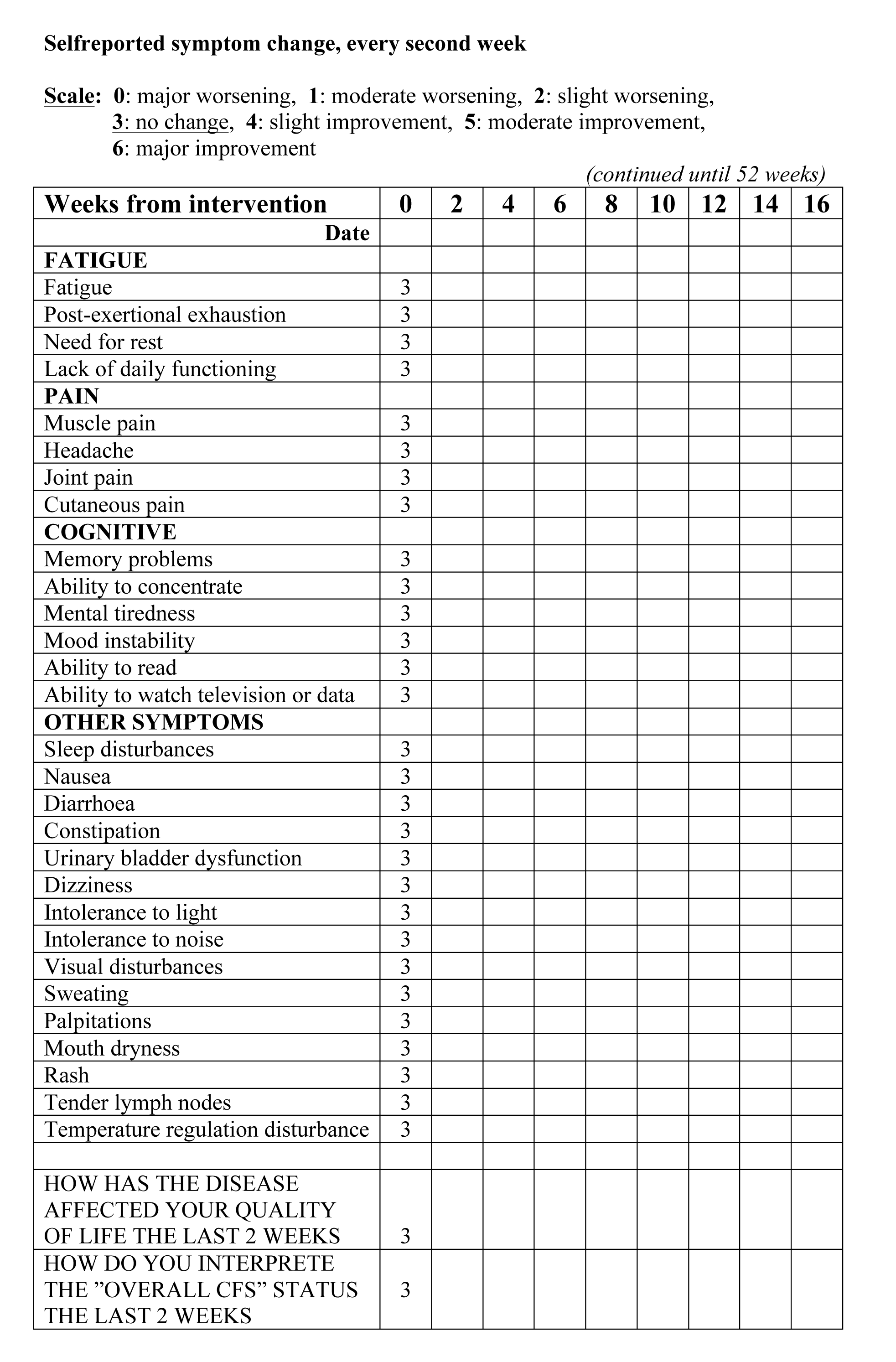

Supplement: Figure S2 — Scheme for patient's self-reported CFS symptom change during follow-up. During 12 months follow-up, the patients recorded symptom changes the preceding two weeks, as compared to baseline. The visual analogue scale for the follow-up scheme was 0–6 (0: Major worsening; 1: Moderate worsening; 2: Slight worsening; 3: No change; 4: Slight improvement; 5: Moderate improvement; 6: Major improvement). (TIF) [file pone.0026358.s003.tif]

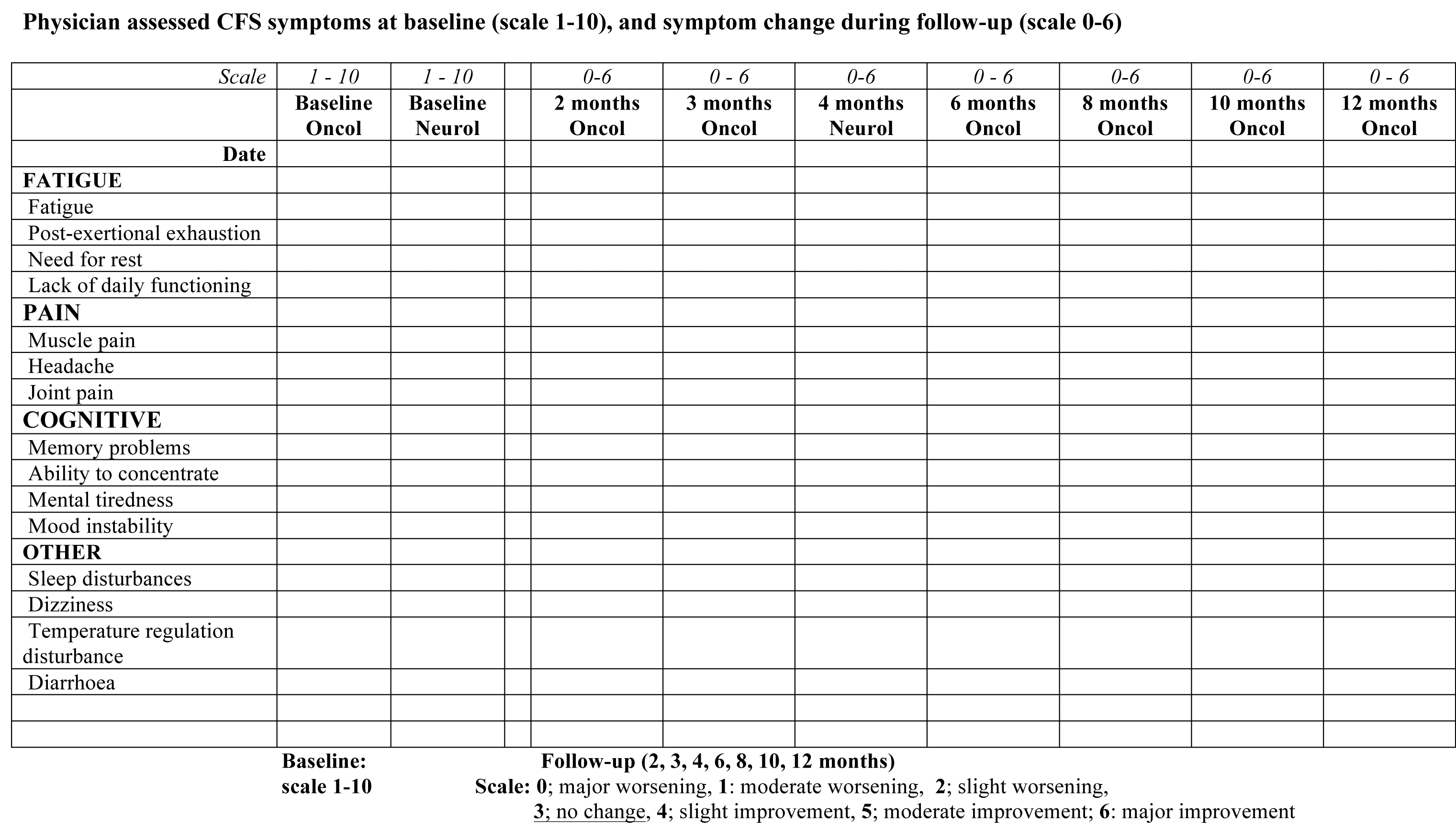

Supplement: Figure S3 — Scheme for physician-assessed CFS symptoms, at baseline and during follow-up. The patients were assessed at the outpatient clinic before intervention, and at 2, 3, 4, 6, 8, 10, and 12 months follow-up. The physicians assessed the patients CFS disease and recorded the symptoms according to visual analogue scales. Before intervention, the scale was 1–10 (1: no symptom; 5: moderate symptom; 10: very severe symptom). During 12 months follow-up, the physicians assessed patients symptom changes as compared to baseline, scale 0–6 (0: Major worsening; 1: Moderate worsening; 2: Slight worsening; 3: No change; 4: Slight improvement; 5: Moderate improvement; 6: Major improvement). (TIF) [file pone.0026358.s004.tif]

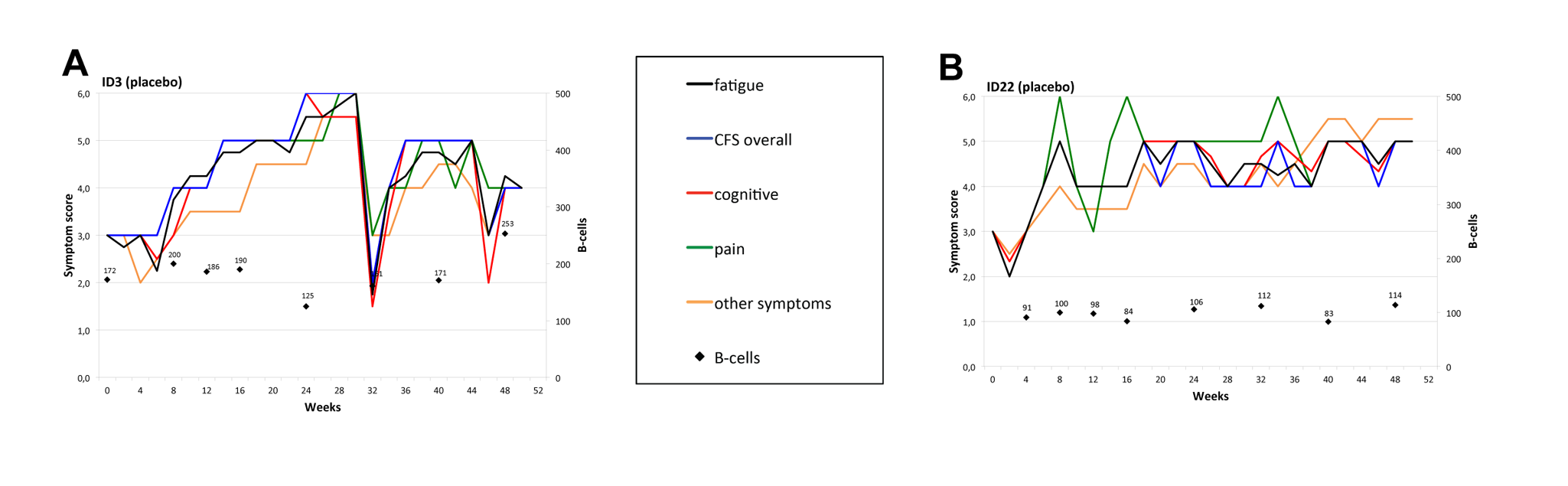

Supplement: Figure S4 — CFS symptom changes during follow-up, for the two patients in the Placebo group with significant improvement. In panels A and B, changes in Fatigue score (black), Cognitive score (red), Pain score (green), “Other symptoms” score (orange), and “CFS overall” score (blue), during 12 months follow-up are shown for the two patients in the Placebo group with significant improvement. The scales on Y-axes were 0–6 (0: Major worsening; 1: Moderate worsening; 2: Slight worsening; 3: No change; 4: Slight improvement; 5: Moderate improvement; 6: Major improvement). Also shown are the B-cell numbers from immunophenotyping of peripheral blood mononuclear cells during follow-up (×106/L). (TIF) [file pone.0026358.s005.tif]
