# Supplementary material for: Benefit from B-Lymphocyte Depletion Using the Anti-CD20 Antibody Rituximab in Chronic Fatigue Syndrome. A Double-Blind and Placebo-Controlled Study
Source: PLoS One. 2011 Oct 19;6(10):e26358. doi: 10.1371/journal.pone.0026358 (PMC3198463; doi:10.1371/journal.pone.0026358)
Supplement: Table S1 — Primers and probes for detection of Xenotropic murine leukemia virus-related virus (XMRV) and MLV-related virus. (PDF) [file pone.0026358.s006.pdf]

Table S1. Primers and probes for detection of Xenotropic murine leukemia virus-related virus (XMRV) and MLV-related virus.

Primer (F/R/P)[1]	Sequence (5' - 3')	Location[2]	Method, Template	Refs.[6]
XMRV4552F (F)<br>XMRV4572MGB (P)<br>XMRV4653R (R)	CGAGAGGCAGCCATGAAGG<br>6FAM-AGTTCTAGAAACCTCTACACTC-MGBNFQ<br>GAGATCTGTTTCGGTGTAATGGAAA	4547-4565<br>4567-4588<br>4624-4648	qPCR<br>gDNA,cDNA<br>viral amp.[4]	Schlaberg
XMRV4552F (F)<br>XMRV4572MGB (P)<br>XMRV4673R (R)	CGAGAGGCAGCCATGAAGG<br>6FAM-AGTTCTAGAAACCTCTACACTC-MGBNFQ<br>CCCAGTTCCCGTAGTCTTTGAG	4547-4565<br>4567-4588<br>4646-4668	qPCR<br>gDNA, cDNA<br>viral amp.[4]	Schlaberg
XMRV_F36 (F)<br>XMRV04-P (P)<br>XMRV_B42 (R)	CCCAAAATCCTGGACCCCTAAG<br>6FAM-GTATCGCTGGACCACGGA-BHQ1<br>CATACCTCTGAAGGCAACCTCTACC	2325-2346<br>2389-2406<br>2725-2749	qPCR<br>gDNA, cDNA	in house
XMRV_04F (F)<br>XMRV_04P (P)<br>XMRV_04R (R)	CTAAGTGACAAGTCTGCC<br>6FAM-GTATCGCTGGACCACGGA-BHQ1<br>CGCAAAGTACATCTGGCT	2342-2359<br>2389-2406<br>2408-2425	qPCR<br>gDNA, cDNA<br>viral amp.[4]	in house
gag 419F (F)<br>gag 1154R (R)	ATCAGTTAACCTACCCGAGTCGGAC<br>GCCGCCTCTTCTTCATTGTTCTC	419-443<br>1127-1149	PCR<br>gDNA	Lombardi
env 5922F (F)<br>env 6273R (R)	GCTAATGCTACCTCCCTCCTGG<br>GGAGCCCACTGAGGAATCAAAACAGG	5917-5938<br>6242-6267	PCR<br>gDNA	Lombardi
gag 419F (F)<br>gag 1154R (R)<br>GAG-I-F (F)<br>GAG-I-R (R)	ATCAGTTAACCTACCCGAGTCGGAC<br>GCCGCCTCTTCTTCATTGTTCTC<br>TCTCGAGATCATGGGACAGA<br>AGAGGGTAAGGGCAGGGTAA	419-443<br>1127-1149<br>598-617<br>991-1012	Nested PCR<br>gDNA	Lombardi<br><br>Urisman
419F (F)<br>1154R (R)<br>NP116F (F)<br>NP117R (R)	ATCAGTTAACCTACCCGAGTCGGAC<br>GCCGCCTCTTCTTCATTGTTCTC<br>CATGGGACAGACCGTAACTACC<br>GCAGATCGGGACGGAGGTTG	419-443<br>1127-1149<br>607-628<br>968-987	Nested PCR<br>gDNA, cDNA<br>viral amp.[4]	Lombardi<br><br>Lo
XMRV_F36 (F)<br>XMRV_B42 (R)<br>XMRV_04F (F)<br>XMRV_04R (R)	CCCAAAATCCTGGACCCCTAAG<br>CATACCTCTGAAGGCAACCTCTACC<br>CTAAGTGACAAGTCTGCC<br>CGCAAAGTACATCTGGCT	2325-2346<br>2725-2749<br>2342-2359<br>2408-2425	Nested PCR<br>gDNA	in house<br><br>in house
GAG-O-F (F)<br>GAG-O-R (R)<br>GAG-I-F (F)<br>GAG-I-R (R)	CGCGTCTGATTTGTTTTGTT<br>CCGCCTCTTCTTCATTGTTC<br>TCTCGAGATCATGGGACAGA<br>AGAGGGTAAGGGCAGGGTAA	537-556<br>1129-1148<br>598-617<br>991-1012	Nested PCR<br>gDNA,cDNA,	Urisman<br><br>Urisman
PMP22ex3F (F)<br>PMP22ex3P (P)<br>PMP22Ex3R (R)	GGGCAATGGACACGCAACT<br>6FAM-CTGGCAGAACTGTAGCACCTCTTCC-BHQ1<br>TGATGAGAAACAGTGGTGGACA	11146-11164[3]<br>11170-11194[3]<br>11203-11224[3]	qPCR<br>gDNA	in house

[1]: F; forward primer, R; reverse primer, P; taqman probe.
[2]: XMRV sequence according to GenBank accession number EF185282.
[3]: According to GenBank accession number NG_007949.1. Peripheral myelin protein 22 (PMP22, a single copy gene) was used to normalize for PCR using gDNA as input. β-actin (assay ID Hs99999903_m1) was used as internal control for RT-PCR.
[4]: Coculture of patient peripheral blood mononuclear cells with LNCaP prostate carcinoma cells for viral amplification prior to PCR, using the primer setups indicated and both gDNA and cDNA as templates. Performed for nine patients.

[6]: References

Schlaberg R, Choe DJ, Brown KR, Thaker HM, Singh IR: XMRV is present in malignant prostatic epithelium and is associated with prostate cancer, especially high-grade tumors. *Proc Natl Acad Sci U S A* 2009, 106(38):16351-16356.

Lombardi VC, Ruscetti FW, Das Gupta J, Pfost MA, Hagen KS, Peterson DL, Ruscetti SK, Bagni RK, Petrow-Sadowski C, Gold B, Dean M, Silverman RH, Mikovits JA: Detection of an Infectious Retrovirus, XMRV, in Blood Cells of Patients with Chronic Fatigue Syndrome. *Science* 2009, 326(5952):585-589.

Urisman A, Molinaro RJ, Fischer N, Plummer SJ, Casey G, Klein EA, Malathi K, Magi-Galluzzi C, Tubbs RR, Ganem D, Silverman RH, DeRisi JL: Identification of a novel Gammaretrovirus in prostate tumors of patients homozygous for R462Q RNASEL variant. *PLoS Pathog* 2006, 2(3):e25.

Lo SC, Pripuzova N, Li B, Komaroff AL, Hung GC, Wang R, Alter HJ: Detection of MLV related virus gene sequences in blood of patients with chronic fatigue syndrome and healthy blood donors. *Proc Natl Acad Sci U S A* 2010, 107(36):15874-15879.
