# Supplementary material for: Benefit from B-Lymphocyte Depletion Using the Anti-CD20 Antibody Rituximab in Chronic Fatigue Syndrome. A Double-Blind and Placebo-Controlled Study
Source: PLoS One. 2011 Oct 19;6(10):e26358. doi: 10.1371/journal.pone.0026358 (PMC3198463; doi:10.1371/journal.pone.0026358)
Supplement: Table S2 — Effects of intervention group (Rituximab versus Placebo) on Fatigue score during 12 months follow-up, using General Linear Model for repeated measures, with separate analyses for self-reported and physician-assessed symptoms. (DOC) [file pone.0026358.s007.doc]

**Table S2**. Effects of intervention group (Rituximab versus Placebo) on *Fatigue score* during 12 months follow-up, using general linear analysis for repeated measures, with separate models for self-reported and physician-assessed symptoms.

|  | **Rituximab** | | | **Placebo** | | | **Time effect (F)** | **Time effect p-value^a^** | **Time * group effect (F)** | | **Time * group effect p-value^a^** |
| --- | --- | --- | --- | --- | --- | --- | --- | --- | --- | --- | --- |
| Self-reported fatiguescore (0 - 6) | N | Mean | SD | N | Mean | SD |  |  |  | |  |
|  |  |  |  |  |  |  | 2.59 | 0.058 | 3.52 | | 0.018 |
| baseline | 15 | 3.00 | 0.00 | 15 | 3.00 | 0.00 |  | | | | |
| 0 – 16 weeks | 15 | 3.20 | 0.39 | 15 | 3.20 | 0.45 |
| 16 – 24 weeks | 15 | 3.56 | 0.85 | 15 | 3.15 | 0.98 |
| 24 – 32 weeks | 15 | 3.82 | 0.69 | 15 | 3.05 | 0.93 |
| 32 – 40 weeks | 15 | 3.70 | 0.92 | 14^b^ | 2.90 | 0.79 |
| 40 – 52 weeks | 15 | 3.48 | 1.02 | 13^b^ | 2.85 | 0.88 |
|  |  |  |  |  |  |  |  |  |  |  | |
| Physician-assessed fatiguescore (0 – 6) | N | Mean | SD | N | Mean | SD |  |  |  |  | |
|  |  |  |  |  |  |  | 3.75 | 0.010 | 3.10 | | 0.024 |
| baseline | 15 | 3.00 | 0.00 | 15 | 3.00 | 0.00 |  | | | | |
| 2 - 4 months | 15 | 3.49 | 0.62 | 15 | 3.36 | 0.67 |
| 6 months | 15 | 4.00 | 1.08 | 15 | 3.31 | 1.00 |
| 8 months | 15 | 3.96 | 1.05 | 15 | 2.98 | 0.61 |
| 10 months | 15 | 3.80 | 1.10 | 14^b^ | 2.96 | 0.84 |
| 12 months | 15 | 3.62 | 1.15 | 13^b^ | 3.00 | 0.61 |

^a^: p-values from Greenhouse-Geisser adjustments, due to significant Mauchly’s test for sphericity.

^b^: Out of 150 symptom registrations, 3 (2%) were missing. One patient was pregnant after 8 months follow-up, and one patient withdrew from study after 10 months follow-up for alternative therapy. For these two patients to be included in the general linear model for repeated measures, the mean values for fatigue scores from the two registrations preceding withdrawal from follow-up were plotted in the missing cells. Both patients with missing data were allocated to the placebo group, and had no sign of clinical response during follow-up.
