# Supplementary material for: Benefit from B-Lymphocyte Depletion Using the Anti-CD20 Antibody Rituximab in Chronic Fatigue Syndrome. A Double-Blind and Placebo-Controlled Study
Source: PLoS One. 2011 Oct 19;6(10):e26358. doi: 10.1371/journal.pone.0026358 (PMC3198463; doi:10.1371/journal.pone.0026358)
Supplement: Protocol S1 — Trial Protocol. (PDF) [file pone.0026358.s008.pdf]

DRUG INTERVENTION IN
CHRONIC FATIGUE SYNDROME (KTS-1-2008)
A RANDOMISED PHASE II STUDY

Protocol approved May 21th, 2008

Amendment approved Jan 22nd, 2009

EudraCT: 2007-007973-22

<u>Contents:</u>

-Protocol
-Project description
-Patient information and consent
-Patient's self-reporting of symptoms
    -before treatment (baseline)
    -every second week during follow-up
    -SF-36 short form (every fourth week)
-Recording by physician
    -scheme for registration before treatment and during follow-up
    -fatigue scale, fatigue severity scale
-Other attachments
    -guidelines for Rituximab infusion
    -labelling of infusion bag

Dept. of Oncology and Medical Physics, and Dept. of Neurology,
Haukeland University Hospital

--------------------- ----------------------- ----------------------

--------------------- ----------------------- ----------------------

PROTOCOL (including amendment)

Protocol approved 21.05.2008

Amendment approved 22.01.2009

DRUG INTERVENTION IN CHRONIC FATIGUE SYNDROME (KTS-1-2008)

A RANDOMISED PHASE II STUDY

EudraCT: 2007-007973-22

STUDY CENTRE

This study is performed at the Dept. of Oncology, Haukeland University Hospital, in collaboration with Dept. of Neurology, Haukeland University Hospital

STUDY PARTICIPANTS

-Olav Mella, director, prof. dr. med, Dept. of Oncology, Haukeland University Hospital (project leader).

-Øystein Fluge, consultant dr. med, Dept. of Oncology, Haukeland University Hospital (project coordinator).

-Harald Nyland, prof. dr. med, Dept. of Neurology, Haukeland University Hospital

-Halvor Næss, consultant, dr. med, Dept. of Neurology, Haukeland University Hospital

-Olav Dahl, prof., dr. med, Dept. of Oncology, Haukeland University Hospital

-Anette Storstein, consultant, Ph.D., Dept. of Neurology, Haukeland University Hospital

BACKGROUND

See project description:

”Drug intervention in chronic fatigue syndrome” (February 2008).

STUDY AIMS AND ENDPOINTS

Chronic fatigue syndrome (CFS)/ Myalgic encephalomyelopathy (ME) is an illness of unknown aetiology, often with major symptoms and severe impairment of daily functioning. As described further in the project description, studies suggest that dysregulation of the immune system, often preceded by an exogenous stimulus such as a viral infection, is a central pathogenetic factor in CFS/ME. Data from the CFS literature are not conclusive, but indicate that deregulation both in T-lymphocytes, NK-cells and B-lymphocytes may be important. There are several clues to an activated B-cell system as part of the pathogenetic mechanism.

Our clinical observations of patients with synchronous lymphoma and CFS, and of pilot patients with CFS, treated either with chemotherapy with immunosuppressive effects or with the B-cell depletion agent Rituximab, support our assumption that B-cell depletion may be therapeutically effective in CFS (see project description).

The hypothesis is that B-cell depletion using the monoclonal anti-CD20 antibody Rituximab (Mabthera®) is associated with improvement of clinical symptoms in CFS, as compared to placebo. In the study, we will also collect biological samples (blood samples) from patients before treatment and during follow-up in order to investigate different laboratory parameters and changes in gene expression in lymphocytes, in patients with CFS treated without active drug (placebo) and in CFS patients treated with Rituximab. These data will be related to clinical responses.

Primary end-point:

-to analyse effects on CFS symptoms, by assessment 3 months after start of two infusions of the monoclonal anti-CD20 antibody Rituximab (Mabthera®) (500 mg/m^2, max 1000 mg per infusion), or placebo (saline), given two weeks apart.

Secondary end-points:

-to analyse effects on CFS symptoms, by assessment at 2, 4, 6, 8, 10 and 12 months after start of two infusions of the monoclonal anti-CD20 antibody Rituximab (Mabthera®) (500 mg/m^2, max 1000 mg per infusion), or placebo (saline), given two weeks apart.

The therapeutic effects at primary and secondary end-points are recorded by patients self-reporting of symptoms and by physician's assessment.

-to evaluate toxicity during 12 months follow-up, after two infusions of the monoclonal anti-CD20 antibody Rituximab (Mabthera®) (500 mg/m^2, max 1000 mg per infusion), or placebo (saline), given two weeks apart.

DESIGN

Double-blind, randomised, phase II, placebo-controlled study. Single centre.

NUMBER OF PATIENTS

Thirty (30) evaluable patients with CFS/ ME.

Fifteen (15) in each arm. Randomised to treatment with Rituximab or Placebo (NaCl 0.9%).

The patients will be identified from those referred to the Dept. of Neurology, Haukeland University Hospital, with ICD-10 code G93.3. These will be contacted and invited to Dept. of Oncology for information and assessment for inclusion in the study. We will try to include patients also with a definite infection preceding CFS onset. We realise that 30 patients is a relatively small number for a randomised phase II study. However, we assume that responders may have marked improvements of CFS symptoms and that we will be able to record significant changes after Rituximab treatment as compared to Placebo.

INCLUSION CRITERIA

-Patients with CFS/ ME, according to revised Fukuda criteria from 1994, including duration of at least six months, and diagnosed by a neurologist.

-Age 18 – 60 years.

-Informed and signed consent.

EXCLUSION CRITERIA

-Patients with fatigue not fulfilling diagnostic criteria for CFS/ ME, or with duration less than six months, or fatigue in which diagnostic procedures suggest other pathology as a cause of the symptoms.

-Age < 18 years, or age > 60 years.

-Pregnancy or lactation. Positive serum or urine pregnancy test.
-Previous malignant disease (excluding basal cell carcinoma in the skin, or cervical dysplasia in the uterus).
-Previous major immunological disease, except common autoimmune diseases such as e.g. thyroiditis or diabetes mellitus type I.
-Previous long-term systemic immunosuppressive medication (Steroids in high doses, Cyclosporine, Mycophenolatmofetil, Azathioprin). Excluding short-lasting steroid courses in e.g. obstructive lung disease.
-Major endogenous depression.
-Lack of ability to adhere to protocol.
-Previous treatment with Rituximab.
-Previous major multi-allergy and assessed to be at risk from Rituximab infusion.
-Reduced renal function (s-creatinine > 1.2 x upper normal value).
-reduced liver function (s-bilirubin > 1.5 x upper normal level, or liver transaminases > 1.5 x upper normal level).
-Known HIV positivity.
-Evidence for active ongoing and relevant viral infection.

DATA MANAGEMENT AND STATISTICAL ANALYSES

The code for intervention (Rituximab versus Placebo) will be revealed at earliest four months after the last patient started intervention. Clinically relevant improvements in CFS symptoms will be defined.
The active treatment group (Rituximab) and Placebo group will be compared for demographic and clinical characteristics. Longitudinal changes in health-related quality of life will be recorded. We will analyse symptom changes for the individual patients and for the patients groups (Rituximab versus Placebo), with emphasis on symptom changes over time. Statistical and clinical significant improvements will be assessed.
The SF-36 (short form 36) scheme will also be used for patients self-reporting of symptoms and has been thoroughly evaluated.
The patients will record baseline levels and self-reported changes in symptom scores (as compared to baseline) every second week on a separate scheme, and SF-36 every month, during 12 months follow-up.

Assessments by the physician will be performed at baseline and after 2, 3, 4, 6, 8, 10 and 12 months. These assessments will be performed at the outpatient clinic (Dept. of Oncology), and symptom changes as compared to baseline are recorded on a separate scheme. Also, schemes for Fatigue Scale and Fatigue Severity Scale are recorded at 2, 3 and 4 months after intervention. All patients will be assessed by two physicians (oncologist and neurologist) at baseline. An oncologist will assess the patients after 2, 3, and 6, 8, 10 and 12 months. A neurologist will assess the patients after 4 months. Toxicity after 6 and 12 months will be assessed by the oncologist. At each visit the actual status of the patient will be compared to baseline (at inclusion, before intervention).

Given the fact that there are no diagnostic laboratory tests or other specific tests for CFS, variables describing the patient's symptom changes will be crucial for adequate assessment. The effect variables will therefore centre on the four main categories of symptoms in CFS: Fatigue, Pain, Cognitive/Mental, and Others (including symptoms from the autonomic nervous system, see scheme for self-reporting of symptoms). The patients will also record (every second week) how they interpreted their overall CFS status, and how they interpreted their total quality of life.

Because the symptoms (within the four main categories) may vary between patients, we will try to define at baseline which symptoms that are characteristic for each patient. Only symptoms that are part of a patient's CFS disease will be analysed (e.g. if skin pain is not relevant for one patient, this symptom will not be recorded during follow-up and analysed for that patient). For the symptom category ”Other symptoms” (see scheme for self-reported symptoms) only those symptoms described at baseline as part of the patient's disease picture will be analysed. The patients will also be evaluated at 6 and 12 months for toxicity. Longitudinal changes in CFS symptoms (within each of the four main categories) over time, as compared to baseline, for each patient and for the intervention group, will be analysed.

The patient's subjective grading of the actual symptom will be recorded at baseline. The changes in the actual symptoms, compared to baseline, will be recorded every second week. The statistical data program SPSS will be used for analyses. Data registration from the schemes and plotting will be performed by Øystein Fluge, and monitored by Olav Mella. External monitoring of the study will be considered and performed if needed.

All schemes with original data will be stored at Dept. of Oncology, Haukeland University Hospital, for 15 years. All data files will also be stored at the Dept. of Oncology. At all visits at the outpatient clinic (Dept. of Oncology) the physicians will make notes in the electronic hospital files.

RANDOMISATION

Randomisation will be performed at The Pharmacy, Haukeland University Hospital, and each patient will be given an inclusion number. The randomisation will be simple, with no blocking, 1:1, and performed prior to study start. The pharmacy will make the infusion bags, with study-specific labelling, and the infusion bags delivered to Dept. of Oncology, Lymphoma section.

The codes for intervention will be stored at the Pharmacy until at least four months after the last patient received intervention (exception for major toxicity or unexpected events). All patients will be unblinded at the same time, at least four months (or later) after the last included patient received intervention.

The patients will be informed about group allocation (Rituximab versus Placebo) at least six months (or later) after the last included patient received intervention, i.e. at least two months (or later) after unblinding. All patients will be informed about group allocation approximately at the same time (period of 1-2 weeks).

If the study should demonstrate a significant therapeutic effect of Rituximab, the patients in the placebo group will be offered participation in a new Phase II one-armed study, using treatment with Rituximab (without a placebo group). This new study will be planned and approvements applied (Regional Ethical Committee, Norwegian Medicines Agency) according to usual procedures.

INFORMATION AND ASSESSMENT AT THE OUTPATIENT CLINIC, DEPT. OF ONCOLOGY

The patients are initially identified from lists at the Dept. of Neurology (ICD-10 code G93.3), and contacted (directly by the neurologist or by telephone). Eligible patients are invited to the Dept. of Oncology, Haukeland University Hospital for thorough assessment, verbal and written information, and written consent.

INVESTIGATIONS AND REGISTRATION AFTER INFORMED WRITTEN CONSENT, BEFORE INCLUSION AND INTERVENTION

Clinical assessment:

-Assessment and registration of CFS/ME symptoms and grading of these.

-Exclusion of other medical causes of major fatigue, such as hypothyreosis, Addison's disease, malignant disease, chronic infections, lung disease, coronary disease, heart failure, renal failure, liver disease, other neurological disease (like multiple sclerosis, brain tumour, cerebrovascular disease), endogenous depression or other psychiatric diseases associated with fatigue.

Laboratory tests:

-Hemoglobin, SR, Leucocytes with differential count, platelets, MCV

-ferritin, s-iron, cobalamin, folate, Na, K, Ca, Mg, phosphate, glucose

-creatinine, carbamid, urat, triglycerides, total cholesterol, HDL cholesterol

-liver transaminases (ALAT, ALP, GT), bilirubin

-C-reactive protein, albumin, total protein, INR

-Pregnancy test (serum) for fertile women

Immunology:

-serum electrophoresis, immunoglobulins (IgG with subclasses, IgM, IgA)

-flowcytometric immunophenotyping of lymphocytes in peripheral blood

-antibodies to transglutaminase (celiac disease), antinuclear antibodies, Rheumatoid factor, thyroid antibodies (anti-TPO, anti-TG), antibodies to adrenal gland

-complement factors C3 and C4

Endocrinology:

-freeT4, TSH, prolactin, cortisol/ACTH

Microbiology:

-Serology for EBV, CMV, HSV, VZV, Enterovirus, Parvovirus B19, adenovirus

Samples for biobank

Results from immunophenotyping of peripheral blood lymphocytes, and from differential counts of leucocytes during follow-up, will not be available to patients or to the physicians performing assessments of patients, until the code for group allocation has been revealed (unblinding of the study).

ADMISSION TO DEPT. OF ONCOLOGY, LYMPHOMA SECTION, FOR INTERVENTION

The assessment of patients and inclusion will be performed before the patients are admitted the Dept. of Oncology for in-hospital intervention (Rituximab or Placebo). The patients randomised to the Rituximab group will receive Rituximab dose 500 mg/m^2, max 1000 mg, dissolved in NaCl 0.9 % to a concentration of 2 mg/ml. Patients randomised to Placebo, will receive an equal volume of NaCl 0.9 %, given according to identical guidelines.

The second infusion will be given 10 – 14 days after the first infusion, with identical dose/volume Rituximab or placebo.

The day of infusion, all patients will receive Cetirizine 10 mg x 1 po, Paracetamol 1 g x 2 po, and Dexamethasone 8 mg x 1 po.

The infusions will follow the guidelines for Rituximab treatment in lymphoma patients, with nurse present and monitoring of blood pressure, pulse and oxygen saturation, as specified. If the first infusion is performed with no symptoms or discomfort, the next infusion (two weeks later) will be given in a shorter time frame, according to the local guidelines for lymphoma treatment.

2 weeks after the first intervention:

Laboratory tests:

Hemoglobin, Leucocytes, platelets, liver transaminases (ALAT, ALP, GT), bilirubin, creatinine, C-reactive protein

4 weeks after first intervention:

Laboratory tests:

Hemoglobin, Leucocytes, platelets, liver transaminases (ALAT, ALP, GT), bilirubin, creatinine, C-reactive protein

INVESTIGATIONS AND REGISTRATION AT 2 MONTHS FOLLOW-UP

Clinical assessment and registration by oncologist.

Copy of patient's self-reported symptom schemes.

Laboratory tests:

-Hemoglobin, SR, leucocytes, platelets, MCV, Na, K, Ca, Mg, phosphate, glucose, creatinine, carbamid, urate, liver transaminases (ALAT, ALP, GT), bilirubin, C-reactive protein, albumin, total protein, INR

Immunology:

-serum electrophoresis, immunoglobulins IgG, IgM, IgA

-immunophenotyping of lymphocytes in peripheral blood.

Samples for biobank.

INVESTIGATIONS AND REGISTRATION AT 3 MONTHS FOLLOW-UP

Clinical assessment and registration by oncologist.

Copy of patient's self-reported symptom schemes.

Laboratory tests:

-Hemoglobin, SR, leucocytes, platelets, MCV, Na, K, Ca, Mg, phosphate, glucose, creatinine, carbamid, urate, liver transaminases (ALAT, ALP, GT), bilirubin, C-reactive protein, albumin, total protein, INR

Immunology:

-serum electrophoresis, immunoglobulins IgG, IgM, IgA

-immunophenotyping of lymphocytes in peripheral blood

Samples for biobank

INVESTIGATIONS AND REGISTRATION AT 4 MONTHS FOLLOW-UP

Clinical assessment and registration by neurologist.

Copy of patient's self-reported symptom schemes.

Laboratory tests:

-Hemoglobin, SR, leucocytes, platelets, MCV, Na, K, Ca, Mg, phosphate, glucose, creatinine, carbamid, urate, liver transaminases (ALAT, ALP, GT), bilirubin, C-reactive protein, albumin, total protein, INR

Immunology:

-serum electrophoresis, immunoglobulins IgG, IgM, IgA

-immunophenotyping of lymphocytes in peripheral blood

Samples for biobank.

INVESTIGATIONS AND REGISTRATION AT 6 MONTHS FOLLOW-UP

Clinical assessment and registration by oncologist

Copy of patient's self-reported symptom schemes

Laboratory tests:

-Hemoglobin, SR, leucocytes, platelets, MCV, Na, K, Ca, Mg, phosphate, glucose, creatinine, carbamid, urate, liver transaminases (ALAT, ALP, GT), bilirubin, C-reactive protein, albumin, total protein, INR

Immunology:

-serum electrophoresis, immunoglobulins IgG, IgM, IgA

-immunophenotyping of lymphocytes in peripheral blood

Samples for biobank.

INVESTIGATIONS AND REGISTRATION AT 8 MONTHS FOLLOW-UP

Clinical assessment and registration by oncologist

Copy of patient's self-reported symptom schemes

Laboratory tests:

-Hemoglobin, SR, leucocytes, platelets, MCV, Na, K, Ca, Mg, phosphate, glucose, creatinine, carbamid, urate, liver transaminases (ALAT, ALP, GT), bilirubin, C-reactive protein, albumin, total protein, INR

Immunology:

-serum electrophoresis, immunoglobulins IgG, IgM, IgA

-immunophenotyping of lymphocytes in peripheral blood

Samples for biobank

INVESTIGATIONS AND REGISTRATION AT 10 MONTHS FOLLOW-UP

Clinical assessment and registration by oncologist.

Copy of patient's self-reported symptom schemes.

Laboratory tests:

-Hemoglobin, SR, leucocytes, platelets, MCV, Na, K, Ca, Mg, phosphate, glucose, creatinine, carbamid, urate, liver transaminases (ALAT, ALP, GT), bilirubin, C-reactive protein, albumin, total protein, INR.

Immunology:

-serum electrophoresis, immunoglobulins IgG, IgM, IgA.

-immunophenotyping of lymphocytes in peripheral blood.

Samples for biobank

INVESTIGATIONS AND REGISTRATION AT 12 MONTHS FOLLOW-UP

Clinical assessment and registration by oncologist.

Copy of patient's self-reported symptom schemes.

Laboratory tests:

-Hemoglobin, SR, leucocytes, platelets, MCV, Na, K, Ca, Mg, phosphate, glucose, creatinine, carbamid, urate, liver transaminases (ALAT, ALP, GT), bilirubin, C-reactive protein, albumin, total protein, INR

Immunology:

-serum electrophoresis, immunoglobulins IgG, IgM, IgA

-immunophenotyping of lymphocytes in peripheral blood

Samples for biobank.

REGISTRATION BY PHYSICIAN AT 0, 2, 3, 4, 6, 8, 10 AND 12 MONTHS FOLLOW-UP

At the outpatient clinic visits the physician makes a clinical assessment of the patient's CFS status, with notes in the electronic hospital files. The schemes for patient's self-reported symptoms are copied.

At each visit, the physician records the assessment of the patient's CFS symptoms on a separate scheme.

REGISTRATION OF PATIENT'S SELF-REPORTED SYMPTOMS

The patient's self-reported CFS symptoms are recorded on a separate scheme, at baseline before intervention, and then every second week during 12 months follow-up. SF-36 schemes are recorded by the patients at baseline, and every month until 10 months follow-up.

Information on the registration schemes

Fatigue severity scale (FSS) is a scheme developed in 1989, including nine statements on fatigue, and the patient's record their degree of agreement (scale 1 – 7) (Krupp et

al 1989; Lerdal et al 2005). FSS in recorded at baseline and at the outpatient clinic visits at 2, 3 and 4 months.
The quality of life SF-36 (short form 36) is used for self-reporting of patient's symptoms, every month during follow-up. SF-36 is a generic (not disease-specific) scheme (Myers & Wilks 1999; Ware & Sherbourne 1992). A Norwegian translation has been validated (Loge et al 1998). Also, the patient's record self-reported symptom changes, as compared to baseline, every second week during 12 months follow-up, on a separate scheme.
The patient's drug consumption is recorded.
Side effects including infections and antibiotic courses are recorded.

TOXICITY
If a SAE (serious adverse event) with possible relation to the intervention should occur, and the medical treatment of this adverse event will require knowledge of intervention group allocation (assessed by project leaders), the code for intervention group for that actual patient will be revealed (by the pharmacy). The procedure will be that the project leader contacts the person at the Pharmacy (Haukeland University Hospital) responsible for the randomisation, for unblinding of intervention code for the actual patient. Serious adverse events, which are unexpected or which may threaten life, will be reported to the Regional Ethical Committee and to the Medicinal Agency within 7 days from the time the study leader has knowledge of the event. Suspected side-effects which are assessed to be serious and unexpected will be reported accordingly, within 15 days. Other side-effects will be collected in a final report. Only SUSARs will be recorded as single reports. The study leaders Øystein Fluge and Olav Mella have the responsibility for reporting to the Regional Ethical Committee and to the Medicinal Agency.
For safety aspects professor Olav Dahl (Dept. of Oncology and Medical Physics, Haukeland University Hospital) will have access to the code for intervention group in case of serious adverse events during nights and weekends. Prof. Olav Dahl will not participate in the clinical assessment of patients.
If any patient is unblinded early due to toxicity or adverse events, data (such as self-reported and physician-assessed CFS symptom changes) from this patient will be analysed for the follow-up period the patient was in blinded design, and data

generated after unblinding will be excluded from analyses. We will however still record possible toxicity for unblinded patients.
The diagnostic procedures prior to inclusion are intended also for exclusion of patients with evidence of active, ongoing and relevant viral infections. Still, the aetiology of CFS is unknown, and it is not possible with absolute certainty to exclude that some patients may have a relevant viral infection not detectable in the present diagnostic procedures, and in such patients B-cell depletion could theoretically worsen the CFS clinical status. We believe that the risk for such worsening is small, but the patients will be informed on this uncertainty both verbally and in the written patient information. As stated in the project description, the toxicity of Rituximab treatment in CFS is at present unknown, and the patients will be followed for 12 months for toxicity recording.

PATIENT WITHDRAWAL DURING FOLLOW-UP

The patients will be informed verbally and in the written patient information that they may withdraw from the study at any time, without having to state the reason for their decision.
The intervention implies two infusions two weeks apart, and we will try to include the planned 30 patients over a relatively short time if possible. If any patient decides to withdraw during the first four weeks after inclusion (”drop-outs”), we will replace these with new patients. The medical reasons for withdrawal from the study may be serious events such as major allergic reactions during or short time after the infusion. Patients who wish to withdraw from the study due to other diseases, or from their own decision, will be followed by their general practitioner, or by the Dept. of Neurology, according to usual guidelines. We will try to obtain data for toxicity also from patients having withdrawn from the study during follow-up.

BIOLOGICAL SPIN-OFF STUDIES

If B-lymphocyte depletion using the anti-CD20 monoclonal antibody Rituximab has beneficial effect in CFS, systematic collection of blood samples may provide a basis for research on the pathogenesis of CFS.
In addition to laboratory tests as specified in this protocol, before intervention and during follow-up after 2, 3, 4, 6, 8, 10 and 12 months, blood samples for research will

be collected at the same time points (after informed written consent from the patients).
The biological spin-off studies will be headed by Olav Mella and Øystein Fluge, and in addition by prof. Olav Dahl (Dept. of Oncology), in collaboration with Ove Bruland (Ph.d., Dept. of Medical Genetics and Molecular Medicine, Haukeland University Hospital), and with prof. Einar K. Kristoffersen (Dept. of Immunology and Transfusion Medicine, Haukeland University Hospital), and with researchers at the Dept. of Oncology (M.Sc. Kristin Risa, Ph.d. Dipak Sapkota).
Samples of serum, plasma, and full blood will be collected at baseline, and after 2, 3, 4, 6, 8, 10, and 12 months follow-up, and stored at -80°C. Samples for purification of total RNA and genomic DNA from peripheral blood mononuclear cells will be collected at baseline, and after 2, 3 and 4 months follow-up, and stored at -80°C. Samples for flowcytometric analyses of lymphocyte subsets will be performed at each visit. Total RNA may be used for global gene expression analyses, with changes in gene expression for selected genes over time during follow-up (Taqman quantitative RT-PCR), both in patients allocated to the placebo group and in patients in the Rituximab group. Data will be analysed also in relation to clinical response. DNA may be used for single nucleotide polymorphism (SNP) analyses. We have not planned studies on possible inheritance in CFS. Other research will be planned according to biological and clinical data generated through the study.

FINANCIAL ASPECTS

The study is initiated by The Dept. of Oncology and Medical Physics, Haukeland University Hospital, and financed by research budgets. There is no external sponsor.

PUBLICATION

Results from the clinical study will be published in a scientific journal, either the result is positive or negative (i.e. no benefit from B-cell depletion in CFS). The Vancouver guidelines will be followed for co-authorships.

APPLICATIONS

We will apply for study approval to the following:

-**The Regional Ethical Committee**

-**The Registry for biobanks** in Norway

-**The Norwegian Data Inspectorate**

-**EudraCT**

-**The Medicinal Agency in Norway**

REFERENCES

(see also project description for extended reference list)

Krupp LB, LaRocca NG, Muir-Nash J, Steinberg AD. 1989. The fatigue severity scale. Application to patients with multiple sclerosis and systemic lupus erythematosus. *Arch Neurol* 46:1121-3

Lerdal A, Wahl A, Rustoen T, Hanestad BR, Moum T. 2005. Fatigue in the general population: a translation and test of the psychometric properties of the Norwegian version of the fatigue severity scale. *Scand J Public Health* 33:123-30

Myers C, Wilks D. 1999. Comparison of Euroqol EQ-5D and SF-36 in patients with chronic fatigue syndrome. *Qual Life Res* 8:9-16

Ware JE, Jr., Sherbourne CD. 1992. The MOS 36-item short-form health survey (SF-36). I. Conceptual framework and item selection. *Med Care* 30:473-83

Loge JH, Kaasa S, Hjermstad MJ, Kvien TK. 1998. Translation and performance of the Norwegian SF-36 Health Survey in patients with rheumatoid arthritis. I. Data quality, scaling assumptions, reliability, and construct validity. *J Clin Epidemiol* 51:1069-76

Williamson RJ, Purcell S, Sterne A, Wessely S, Hotopf M, Farmer A, et al. 2005. The relationship of fatigue to mental and physical health in a community sample. *Soc Psychiatry Psychiatr Epidemiol* 40:126-32

Project description (February 2008)

Drug intervention in Chronic Fatigue Syndrome.
A randomized, phase II study.

Chronic Fatigue Syndrome (CFS)/ Myalgic Encephalomyelitis (ME) was defined in 1988 (Holmes et al 1988), with revised criteria in 1994 (Fukuda et al 1994). CFS is a clinically defined condition, and the diagnosis is dependent on proper medical assessment and exclusion of other physical or psychiatric disorders associated with fatigue. The main criterion is unexplained, severe fatigue, with a defined onset, persisting for at least six consecutive months, and which is accentuated by physical activity. The fatigue results in a substantial reduction of previous levels of occupational, social, and personal activities and often results in disability.

CFS is also characterized by persistent or recurrent occurrence of symptoms such as impairment of short-term memory or concentration, muscle pain, joint pain without evidence of arthritis, headache, sleep disturbances and post-exercise exhaustion (Fukuda et al 1994). Many patients have symptoms from the autonomic nervous system, like disturbed temperature regulation, orthostatic hypotension, nausea or irritable bowel. Although many studies have shown subtle alterations in diverse blood tests or radiological investigations, no biomarker or diagnostic test exists.

The prevalence of CFS worldwide is thought to be 0.4-1.0 %, and the female : male ratio is 3 : 1 (Wyller 2007). The entity is seen both in children and grown-ups, with most spontaneous healing in children. CFS is a disease with major impact on health economy, as most of the patients are relatively young, but have no ability to work and are vigorous users of health care services. Consequences for the health and society economy are thus major.

AETIOLOGY OF CFS

The aetiology and pathogenesis of CFS is unknown. The various hypotheses include immunological, virological, neuroendocrinological, and psychological mechanisms. The pathogenesis of CFS is presumed to be multifactorial and to involve both host

and environmental factors. In a recent review, describing current research priorities in CFS, the urgent need to elucidate the pathogenesis and aetiology is highlighted (Kerr et al 2007a). A collaborative study group was formed in 2001, to investigate the molecular mechanisms of CFS, with the aims to develop a diagnostic test and also to guide the development of more specific treatment.

Gene alterations

Several gene expression studies have been performed in CFS, indicating that there are specific but complex gene alterations in accordance with a dysfunction in immune response and in defence mechanisms. One microarray study showed differential expression of 16 genes in CFS, suggesting T-cell activation and a disturbance of neuronal and mitochondrial function (Kaushik et al 2005). Another microarray study using serial samples of peripheral blood mononuclear cells total RNA, from patients developing CFS after Epstein Barr virus (EBV) infection and also from subjects with EBV infection without development of fatigue, concluded that several genes affecting mitochondrial function and cell cycle were deregulated (Vernon et al 2006). Another gene expression study in CFS suggested disturbance of exercise responsive genes, including several involved in membrane transport and ion channels (Whistler et al 2005). Recently, an analysis of gene networks in CFS revealed seven distinct genomic subtypes with differences in clinical presentation and severity (Kerr et al 2007b). Several other studies have addressed global gene expression in CFS (Fang et al 2006; Whistler et al 2003). The gene expression data are not conclusive, but suggest that there are gene expression disturbances in CFS representing various cellular functions, and may indicate that the disease has a heterogeneous pathogenesis.

Immune deregulation

A prevailing theme in CFS research has been a sustained immune deregulation following acute exogenous stimuli, such as viral infections. Among the microbial pathogens reported to be associated with CFS are Epstein-Barr virus (Lerner et al 2004), enterovirus (Chia & Chia 2007), parvovirus B19 (Matano et al 2003), cytomegalovirus (Lerner et al 2002), human herpesvirus type 6 (Chapenko et al 2006; Komaroff 2006), and Chlamydia pneumoniae (Nicolson et al 2003). However, the data are not consistent (Soto & Straus 2000).

A recent study of postinfective fatigue syndrome found no differences in ex vivo cytokine production over a 12-month period, as compared to controls recovering promptly after infection (Vollmer-Conna et al 2007). Others claim that despite evidence of immune activation, as demonstrated by increased number of activated T-cells and elevated levels of cytokines, the CFS patients may have a reduced immune cell function with a low NK-cell cytotoxicity and immunoglobulin deficiencies (Patarca 2001).

In one study comparing CFS patients and controls, a high number of circulating B-lymphocytes and altered NK-cells subsets, also with increased expression of adhesion molecules, were reported (Tirelli et al 1994). Another study showed reduced CD56+ NK-cells, and reduced CD4+ and CD8+ T-lymphocytes in CFS patients (Racciatti et al 2004). Also, T- and NK-cells from CFS patients were found to express lower levels of the intracellular granule protein perforin, indicating a reduced ability to mediate cytotoxicity (Maher et al 2005).

One study showed several abnormalities in laboratory markers associated with immune function in CFS patients (Klimas et al 1990). The most consistent result was a low NK cell cytotoxicity, but also an increase in CD8+ T-cells, elevated number of CD20+ B-cells, and increase in the B-cell subset coexpressing CD20 and CD5 (Klimas et al 1990). These data were to some extent supported by a study reporting expansion of activated CD8+ cytotoxic T lymphocytes, along with a marked decrease in NK cell activity, in CFS patients (Barker et al 1994).

A recent study comparing CFS patients and controls reported decreased expression of CD69 on T-cells and NK-cells after mitogenic stimulation *in vitro,* indicating a disorder in the early activation of cellular immunity mediated by these cells (Mihaylova et al 2007).

However, the data on immune deregulation in CFS are not consistent, and a study comparing lymphocyte subsets in CFS patients to those of patients with depression, multiple sclerosis and healthy controls, found no difference in T-, B-, or NK-cell subsets (Robertson et al 2005). Similarly, a review of the immunology in CFS concluded that the studies performed in the research field had varying quality, and

that no consistent pattern of immunological abnormalities could be identified (Lyall et al 2003).

Along with hypotheses of immune deregulation in CFS, autoimmunity to endogenous vasoactive neuropeptides has been proposed as a mechanism for the disease (Staines 2005), however presently not supported by scientific data, and autoantibodies to these neuropeptides have not been detected in CFS. One study investigated the presence of circulating anti-muscle and anti-CNS antibodies in CFS patients and controls, with no detected pathogenic antibodies (Plioplys 1997). Another report of antinuclear autoantibodies in CFS concluded that there was no association (Skowera et al 2002), while one study investigating common autoantibodies and antibodies to neuron specific antigens showed higher rates of antibodies to microtubule-associated protein 2 and ssDNA in CFS (Vernon & Reeves 2005). A single study showed the presence of autoantibodies to muscarinic cholinergic receptor in a subset of CFS patients (Tanaka et al 2003), and higher levels of autoantibodies to insoluble cellular antigens were reported in CFS as compared to controls (von Mikecz et al 1997).
At present, there is no direct evidence with consistent data for the presence of pathogenic autoantibodies, or for T-lymphocyte-mediated autoimmunity. No indirect evidence has recreated the CFS disease in an animal model by immunization with antigens analogous to (putative) human autoantigens.

Other hypotheses

Other hypotheses for CFS pathogenesis are blood platelet dysfunction (Kennedy et al 2006), neurological (Natelson et al 2005; Yoshiuchi et al 2006), neuroendocrine (Van Den Eede et al 2007), metabolic or autonomic disturbances, ion channel dysfunction (Chaudhuri et al 2000), zinc deficiency (Maes et al 2006), and toxin exposure or prior vaccinations (Appel et al 2007). Others have focused on an abnormal response to exercise with intracellular immune deregulation as a possible mechanism in CFS pathogenesis (Nijs et al 2004). Also, post-infective impairment of the ability to synthesise n-3 and n-6 long-chain polyunsaturated fatty acids has been proposed as important in the pathophysiology of CFS (Puri 2007).

As can be derived from the above, aetiology and pathogenesis in CFS is presently not clarified.

CURRENT TREATMENT OF CFS

Due to the lack of knowledge of the exact pathogenesis, and with no known causal mechanism, there is no current standard specific treatment for CFS. A systematic review concluded that CFS should be associated with a "biopsychosocial model", with emphasis on progressive muscular rehabilitation, combined with behavioural and cognitive treatment (Maquet et al 2006). The unknown aetiology of CFS is probably the reason for the remarkably few studies performed, evaluating therapy based upon a biological hypothesis.

As the majority of evidence suggests an immune system deregulation, probably triggered by an exogenous stimulus such as a viral infection, two studies have assessed use of intravenous gammaglobulin for CFS. One was a case report in three patients with CFS following an acute parvovirus B19 infection, treated with 5-days intravenous immunoglobulin, with improvement of clinical symptoms and resolution of cytokine dysregulation (Kerr et al 2003). In a double-blinded, placebo-controlled, randomized study of 71 adolescents with CFS, three infusions of gammaglobulin were given one month apart, with functional improvement in the gammaglobulin-treated group at six-month follow-up with average duration 18 months. In the first six months of the trial, both the placebo group and the gammaglobulin-treated group reported improvement (Rowe 1997).

In a pilot study reported in abstract form (Lamprecht 2001), six patients with CFS were given etanercept (Enbrel®, i.e. human tumor necrosis factor receptor p75 Fc fusion protein, which is a soluble competitive TNF-α receptor acting to inhibit the TNF-α mediated cellular response) and a clinical benefit was reported. Among other therapeutic strategies, valganciclovir was used to treat 12 patients with long-standing fatigue and elevated antibody-titres to Epstein-Barr virus or human herpes virus-6. Nine had improvement of the symptoms, however, with uncertainty as to whether the effects were mediated through anti-viral effect or through immunomodulation (Kogelnik et al 2006). Treatment with azithromycin, an antibiotic with immunomodulating properties, gave improvement in 59 % of 99 CFS patients (Vermeulen & Scholte 2006).

In a recent review of current research priorities in CFS (Kerr et al 2007a), new studies are encouraged to focus on the understanding of the molecular pathogenesis of the disease, to test useful biomarkers, and to aid in the development of specific treatment. Various molecular techniques are available and have been used for this purpose, including global gene expression analyses using microarrays (Kerr et al 2007a).

At the Department of Oncology and Medical Physics, Haukeland University Hospital, we have observed a striking symptomatic improvement after cytotoxic chemotherapy in a 43 year old female patient with stable CFS (debut in 1997, preceded by Epstein-Barr infection) who developed Hodgkin's disease (2003). In addition to the cytotoxic effects, the chemotherapeutics given also had an immunomodulatory effect, with short lasting depletion of leukocytes. The marked improvement in fatigue was contrary to what we had expected in a CFS patient, as they generally tolerate all types of drugs and stress poorly. The changes were not interpreted as related to lymphoma activity, and the effects lasted for approximately 5 months, thereafter there was gradual relapse of CFS-symptoms.

When reviewing the literature on CFS in attempts to understand what our patient encountered during and after cancer chemotherapy, we came to the conclusion that modification of the immune system seemed a likely explanation of the marked, but transient symptom improvement she experienced. It may well be that chronic B-cell activation seen in CFS patients is important for the symptoms and also physiological changes reported, such as central nervous blood circulation alterations (Yoshiuchi et al 2006), and reports of lymphocyte infiltration in brain tissue, spinal nerve roots or cardiac muscle.

Possible modes of action of B-cell depletion could be at several sites, such as interaction with the T-cell system thus modifying inflammatory processes, and in influencing the levels of important pleiotropic players in the immune homeostasis, such as the vasoactive neuropeptides (Gonzalez-Rey & Delgado 2005). These have a wide range of activities in the central nervous system. Taking these existing data into account, together with the unexpected improvement of fatigue and pain in our CFS patient after immunomodulatory cytotoxic therapy, we suggest that B-cell depletion

as a concept should be explored in CFS. At present, achievement of this is most readily achieved by the use of the monoclonal anti-CD20 antibody Rituximab.

RITUXIMAB IN B-CELL LYMPHOMA AND AUTOIMMUNITY

Rituximab (Mabthera®) is a monoclonal antibody directed against an epitope in the extracellular portion of the transmembrane molecule CD20. The antibody is a chimeric human-mouse in which the fragment antigen binding (Fab) part is mouse and the Fc-part is human. The CD20 protein is expressed on B-lymphocytes, but not on stem cells or on the mature plasma cells. CD20 is also expressed on the vast majority of B-cell lymphomas. CD20 is implicated in regulation of transmembrane calcium conductance and cell cycle progression, but the precise function is unknown (Janas et al 2005). Upon binding of Rituximab to CD20, an immunological cell killing is mediated through the binding of complement to the Fc part with activation of the complement cascade, and also through activation antibody-dependent cellular cytotoxicity (ADCC) (Glennie et al 2007). The molecule does not internalize or shed from the plasma membrane after Rituximab binding, which allows the monoclonal antibody to persist on the cell surface to extend the immunological attack.

The role of Rituximab in treatment of B-cell lymphomas has emerged rapidly. Immunochemotherapy using Rituximab in combination with chemotherapy, or Rituximab monotherapy in indolent lymphomas, are now current standards of treatment and has improved overall survival in the most common type of aggressive B-cell lymphomas (diffuse large B-cell lymphoma), both in elderly (Coiffier et al 2002) and in younger patients (Pfreundschuh et al 2006), and also in the most common indolent lymphoma (follicular lymphoma) (Marcus et al 2005). In selected patients with follicular lymphoma, Rituximab is also used as maintenance treatment after induction therapy, with infusions every third month for two years, showing improved overall survival (van Oers et al 2006).

In recent years, Rituximab was proved to be an effective treatment also in autoimmune diseases, where the B-cell depletion is often associated with a clinical improvement, e.g. in rheumatoid arthritis (Dass et al 2006). The list of different autoimmune diseases in which Rituximab has a therapeutic role is growing (Sanz et al

2007). For future B-cell targeting and depletion, the development of antibodies to specific B-cell subsets will be important (Dorner & Lipsky 2007).

SAFETY PROFILE OF RITUXIMAB

The safety profile of Rituximab in treatment of B-cell lymphomas is well known and based on experience from a database with in excess of 370 000 patients (Kavanaugh 2006). In lymphoma treatment, mild-to moderate reaction during the first infusion is the most common side-effect, caused by cytokine release primarily in patients with a high initial tumour burden (Solal-Celigny 2006). Allergic reactions may be seen during the infusion, due to the protein nature of the Rituximab molecule. A concern with all B-cell directed therapy is the anticipated effects on humoral immunity. With extended treatment, and in particular with maintenance treatment, i.e. infusions every third month for two years (after induction therapy with perhaps 6-8 Rituximab-infusions every third week), the B-cell depletion is more pronounced and most patients will develop hypogammaglobulinemia. However, the low levels of immunoglobulins and B-cell depletion do not seem to have a major impact on the clinical risk of infections.

One potential serious side effect from use of Rituximab is the development of interstitial lung disease. This is a potentially life-threatening complication, but very rarely reported, with only 16 cases reported in the literature (Wagner et al 2007).

At the Department of Oncology and Medical Physics, Haukeland University Hospital, we have experience from almost daily use of Rituximab for several years. Although encountering several cytokine release reactions and a few allergic reactions, we have not observed acute life-threatening reactions, nor any major vulnerability to infections thought to be caused by Rituximab. Generally, we believe that Rituximab in lymphoma patients is a safe and well tolerated therapy, with low short-term and long-term toxicity.

Safety issues related to Rituximab-treatment in chronic autoimmune diseases are exploited in clinical studies (Edwards et al 2006), but with limited follow-up time so far. The long-term safety therefore remains to be clarified, especially when Rituximab is to be given once or twice yearly for many years. For patients with autoimmune diseases, Rituximab infusions are often given twice (a few weeks apart), and this

sequence may be repeated after 6-12 months, i.e. considerably lower accumulated doses than in lymphoma patients (in the short term).

In CFS, the safety of rituximab treatment is unknown. Given the unknown aetiology of the disease, and possibility that some patients may have an ongoing active chronic viral infection, one might speculate that B-cell depletion could increase the disease activity. However, we believe that in the vast majority of patients the syndrome is caused by a sustained immune dysregulation without ongoing infection.

HYPOTHESIS

Current research and knowledge, and reported therapeutic experiments in few patients (intravenous gammaglobulin, etanercept) suggest that immunomodulatory therapy may have therapeutic efficacy in CFS. The published data in the research field are not altogether consistent, and laboratory tests associated with immune function show varying abnormalities, as described above. Some studies have indicated dysregulation also in the B-cell compartment. Given the central function of B-cells, both in humoral immunity, but also in the close cooperation with the cellular part of the immune system, we believe that B-cell depletion could alter the immune deregulation in a beneficial direction, with subsequent improvement in the clinical symptoms.

As a first pilot patient, we recently gave the above-mentioned woman with previous lymphoma Rituximab 500 mg/m^2 as a single infusion, after informing of the experimental nature of the procedure and the risks involved. Starting six weeks after the infusion, she experienced a marked improvement in symptoms, with markedly less fatigue, decreasing muscle pain, decreasing (intense) burning pain in the skin, and declining headache, accompanied with decreased need for opioid analgesics. Ten weeks after the Rituximab infusion she reported ongoing gradual improvement, which she had not experienced in the course of CFS except for the period following cytotoxic chemotherapy. She rested markedly less, functioned better as a mother and housewife, could take walks, go shopping and do indoor tasks that she in no way could three months ago. Her skin and muscle pain was markedly less and the analgetic drug dosage was reduced to 1/3. Her concentration ability increased significantly, she could watch TV and perform on a computer much better than

before. She may not necessarily be representative of CFS patients, as she has had Hodgkin's disease (no relapse of the lymphoma presently). Nevertheless, Rituximab treatment has had a marked effect on the CFS symptoms, which she has not had since encountering the disease in 1997, with the exception of the 5-month period during cancer chemotherapy. However, 14 weeks after the Rituximab treatment, she again seemed to have gradual symptom recurrence.

We now have treated two additional pilot patients with CFS of several years duration following infectious episodes. Patient 2 had been out of work for several years and barely could walk outside the house to fetch mail in the post-box. He started 4-6 weeks after Rituximab to encounter a gradual and marked improvement of all symptoms of CFS. First diarrhoea improved, thereafter there was less fatigue and loss of energy, so he had taken a trip skiing. He started changing roof tiles and had done work at home that he had been able to perform for 5 years. The greatest change was, however, improvement in cognitive and memory functions. During his disease, he has only been able to read a few pages and has had problems understanding the texts. During the last weeks he has read several books without interruption and has again been able to use a computer and perform computer games. His muscle pain is markedly better. Sound tolerance is increased. At 10 weeks after after infusion the patient reports an obvious effect on the disease and the quality also of his family life.

Pilot patient 3 reported somewhat less symptoms of CFS (precipitated by infection 7 years previously) than the patients 1 and 2 and reported less cognitive symptoms. Four to five weeks after treatment she noted regression of tender lymph nodes in the neck and axilla, normalizing of diarrhoea, less muscle pain, and slight improvement in energy. She thus reports a significant, but rather modest effect on the disease after 10 weeks of observation.

There has not been any unexpected toxicity after treating the first three patients. The only episode noted is a herpes zoster episode in patient 1 occuring 21 weeks after treatment. She has been heavily pretreated with cytotoxic chemotherapy for lymphoma, including high dose chemotherapy with autologous stem cell transplantation nearly 2 years ago.

CLINICAL STUDY

We plan to do a placebo-controlled, double-blinded, single-center, randomized study in collaboration with Department of Neurology, Haukeland University Hospital.
We will include 30 patients with CFS according to strict criteria and after investigation by a neurologist. They will be randomized, in a double-blind fashion, to either treatment with placebo (saline) or Rituximab infusion (500 mg/m^2 mg intravenously) given on day 0 and 14. We plan to make a detailed registration of CFS-related symptoms, both self-reported and assessed by the physician, before treatment and at regular intervals during follow-up for 12 months, as described in the protocol. We will obtain approvements from all the relevant governmental authorities, including the Regional Committee for Research Ethics.

BIOLOGICAL SPIN-OFF STUDIES

Given a significant therapeutic effect at least in a subgroup of CFS patients, the study mentioned above will be suitable for investigation of the pathogenesis of CFS. We will after getting informed consent obtain a broad spectrum of blood samples before and after therapy at regular intervals, also including lymphocyte subgroup separation by flowcytometry, for purified RNA of lymphocyte subsets and microarray analyses for gene expression changes during therapy. We plan to do global gene expression analysis with longitudinal data connected to the treatment and possible responses (see protocol).

REFERENCES

Holmes GP, Kaplan JE, Gantz NM, Komaroff AL, Schonberger LB, Straus SE, et al. 1988. Chronic fatigue syndrome: a working case definition. *Ann Intern Med* 108:387-9

Fukuda K, Straus SE, Hickie I, Sharpe MC, Dobbins JG, Komaroff A. 1994. The chronic fatigue syndrome: a comprehensive approach to its definition and study. International Chronic Fatigue Syndrome Study Group. *Ann Intern Med* 121:953-9

Wyller VB. 2007. The chronic fatigue syndrome--an update. *Acta Neurol Scand Suppl* 187:7-14

Kerr JR, Christian P, Hodgetts A, Langford PR, Devanur LD, Petty R, et al. 2007a. Current research priorities in chronic fatigue syndrome/myalgic encephalomyelitis: disease mechanisms, a diagnostic test and specific treatments. *J Clin Pathol* 60:113-6

Kaushik N, Fear D, Richards SC, McDermott CR, Nuwaysir EF, Kellam P, et al. 2005. Gene expression in peripheral blood mononuclear cells from patients with chronic fatigue syndrome. *J Clin Pathol* 58:826-32

Vernon SD, Whistler T, Cameron B, Hickie IB, Reeves WC, Lloyd A. 2006. Preliminary evidence of mitochondrial dysfunction associated with post-infective fatigue after acute infection with Epstein Barr virus. *BMC Infect Dis* 6:15

Whistler T, Jones JF, Unger ER, Vernon SD. 2005. Exercise responsive genes measured in peripheral blood of women with chronic fatigue syndrome and matched control subjects. *BMC Physiol* 5:5

Kerr J, Burke B, Petty R, Gough J, Fear D, David M, et al. 2007b. Seven genomic subtypes of Chronic Fatigue Syndrome / Myalgic Encephalomyelitis (CFS/ME): a detailed analysis of gene networks and clinical phenotypes. *J Clin Pathol*

Fang H, Xie Q, Boneva R, Fostel J, Perkins R, Tong W. 2006. Gene expression profile exploration of a large dataset on chronic fatigue syndrome. *Pharmacogenomics* 7:429-40

Whistler T, Unger ER, Nisenbaum R, Vernon SD. 2003. Integration of gene expression, clinical, and epidemiologic data to characterize Chronic Fatigue Syndrome. *J Transl Med* 1:10

Lerner AM, Beqaj SH, Deeter RG, Fitzgerald JT. 2004. IgM serum antibodies to Epstein-Barr virus are uniquely present in a subset of patients with the chronic fatigue syndrome. *In Vivo* 18:101-6

Chia JK, Chia AY. 2007. Chronic fatigue syndrome is associated with chronic enterovirus infection of the stomach. *J Clin Pathol*

Matano S, Kinoshita H, Tanigawa K, Terahata S, Sugimoto T. 2003. Acute parvovirus B19 infection mimicking chronic fatigue syndrome. *Intern Med* 42:903-5

Lerner AM, Beqaj SH, Deeter RG, Fitzgerald JT. 2002. IgM serum antibodies to human cytomegalovirus nonstructural gene products p52 and CM2(UL44 and UL57) are uniquely present in a subset of patients with chronic fatigue syndrome. *In Vivo* 16:153-9

Chapenko S, Krumina A, Kozireva S, Nora Z, Sultanova A, Viksna L, et al. 2006. Activation of human herpesviruses 6 and 7 in patients with chronic fatigue syndrome. *J Clin Virol* 37 Suppl 1:S47-51

Komaroff AL. 2006. Is human herpesvirus-6 a trigger for chronic fatigue syndrome? *J Clin Virol* 37 Suppl 1:S39-46

Nicolson GL, Gan R, Haier J. 2003. Multiple co-infections (Mycoplasma, Chlamydia, human herpes virus-6) in blood of chronic fatigue syndrome patients: association with signs and symptoms. *Apmis* 111:557-66

Soto NE, Straus SE. 2000. Chronic Fatigue Syndrome and Herpesviruses: the Fading Evidence. *Herpes* 7:46-50

Vollmer-Conna U, Cameron B, Hadzi-Pavlovic D, Singletary K, Davenport T, Vernon S, et al. 2007. Postinfective fatigue syndrome is not associated with altered cytokine production. *Clin Infect Dis* 45:732-5

Patarca R. 2001. Cytokines and chronic fatigue syndrome. *Ann N Y Acad Sci* 933:185-200
Tirelli U, Marotta G, Improta S, Pinto A. 1994. Immunological abnormalities in patients with chronic fatigue syndrome. *Scand J Immunol* 40:601-8
Racciatti D, Dalessandro M, Delle Donne L, Falasca K, Zingariello P, Paganelli R, et al. 2004. Study of immune alterations in patients with chronic fatigue syndrome with different etiologies. *Int J Immunopathol Pharmacol* 17:57-62
Maher KJ, Klimas NG, Fletcher MA. 2005. Chronic fatigue syndrome is associated with diminished intracellular perforin. *Clin Exp Immunol* 142:505-11
Klimas NG, Salvato FR, Morgan R, Fletcher MA. 1990. Immunologic abnormalities in chronic fatigue syndrome. *J Clin Microbiol* 28:1403-10
Barker E, Fujimura SF, Fadem MB, Landay AL, Levy JA. 1994. Immunologic abnormalities associated with chronic fatigue syndrome. *Clin Infect Dis* 18 Suppl 1:S136-41
Mihaylova I, DeRuyter M, Rummens JL, Bosmans E, Maes M. 2007. Decreased expression of CD69 in chronic fatigue syndrome in relation to inflammatory markers: evidence for a severe disorder in the early activation of T lymphocytes and natural killer cells. *Neuro Endocrinol Lett* 28:477-83
Robertson MJ, Schacterle RS, Mackin GA, Wilson SN, Bloomingdale KL, Ritz J, et al. 2005. Lymphocyte subset differences in patients with chronic fatigue syndrome, multiple sclerosis and major depression. *Clin Exp Immunol* 141:326-32
Lyall M, Peakman M, Wessely S. 2003. A systematic review and critical evaluation of the immunology of chronic fatigue syndrome. *J Psychosom Res* 55:79-90
Staines DR. 2005. Do vasoactive neuropeptides and heat shock proteins mediate fatigue-related autoimmune disorders? *Med Hypotheses* 64:539-42
Plioplys AV. 1997. Antimuscle and anti-CNS circulating antibodies in chronic fatigue syndrome. *Neurology* 48:1717-9
Skowera A, Stewart E, Davis ET, Cleare AJ, Unwin C, Hull L, et al. 2002. Antinuclear autoantibodies (ANA) in Gulf War-related illness and chronic fatigue syndrome (CFS) patients. *Clin Exp Immunol* 129:354-8
Vernon SD, Reeves WC. 2005. Evaluation of autoantibodies to common and neuronal cell antigens in Chronic Fatigue Syndrome. *J Autoimmune Dis* 2:5
Tanaka S, Kuratsune H, Hidaka Y, Hakariya Y, Tatsumi KI, Takano T, et al. 2003. Autoantibodies against muscarinic cholinergic receptor in chronic fatigue syndrome. *Int J Mol Med* 12:225-30
von Mikecz A, Konstantinov K, Buchwald DS, Gerace L, Tan EM. 1997. High frequency of autoantibodies to insoluble cellular antigens in patients with chronic fatigue syndrome. *Arthritis Rheum* 40:295-305
Kennedy G, Norris G, Spence V, McLaren M, Belch JJ. 2006. Is chronic fatigue syndrome associated with platelet activation? *Blood Coagul Fibrinolysis* 17:89-92
Natelson BH, Weaver SA, Tseng CL, Ottenweller JE. 2005. Spinal fluid abnormalities in patients with chronic fatigue syndrome. *Clin Diagn Lab Immunol* 12:52-5
Yoshiuchi K, Farkas J, Natelson BH. 2006. Patients with chronic fatigue syndrome have reduced absolute cortical blood flow. *Clin Physiol Funct Imaging* 26:83-6

Van Den Eede F, Moorkens G, Van Houdenhove B, Cosyns P, Claes SJ. 2007. Hypothalamic-pituitary-adrenal axis function in chronic fatigue syndrome. *Neuropsychobiology* 55:112-20
Chaudhuri A, Watson WS, Pearn J, Behan PO. 2000. The symptoms of chronic fatigue syndrome are related to abnormal ion channel function. *Med Hypotheses* 54:59-63
Maes M, Mihaylova I, De Ruyter M. 2006. Lower serum zinc in Chronic Fatigue Syndrome (CFS): relationships to immune dysfunctions and relevance for the oxidative stress status in CFS. *J Affect Disord* 90:141-7
Appel S, Chapman J, Shoenfeld Y. 2007. Infection and vaccination in chronic fatigue syndrome: myth or reality? *Autoimmunity* 40:48-53
Nijs J, De Meirleir K, Meeus M, McGregor NR, Englebienne P. 2004. Chronic fatigue syndrome: intracellular immune deregulations as a possible etiology for abnormal exercise response. *Med Hypotheses* 62:759-65
Puri BK. 2007. Long-chain polyunsaturated fatty acids and the pathophysiology of myalgic encephalomyelitis (chronic fatigue syndrome). *J Clin Pathol* 60:122-4
Maquet D, Demoulin C, Crielaard JM. 2006. Chronic fatigue syndrome: a systematic review. *Ann Readapt Med Phys* 49:337-47, 418-27
Kerr JR, Cunniffe VS, Kelleher P, Bernstein RM, Bruce IN. 2003. Successful intravenous immunoglobulin therapy in 3 cases of parvovirus B19-associated chronic fatigue syndrome. *Clin Infect Dis* 36:e100-6
Rowe KS. 1997. Double-blind randomized controlled trial to assess the efficacy of intravenous gammaglobulin for the management of chronic fatigue syndrome in adolescents. *J Psychiatr Res* 31:133-47
Lamprecht P. 2001. Pilot study of etanercept treatment in patients with chronic fatigue syndrome. . *Meeting of the American association of chronic fatigue syndrome (AACFS). Seattle.*
Kogelnik AM, Loomis K, Hoegh-Petersen M, Rosso F, Hischier C, Montoya JG. 2006. Use of valganciclovir in patients with elevated antibody titers against Human Herpesvirus-6 (HHV-6) and Epstein-Barr Virus (EBV) who were experiencing central nervous system dysfunction including long-standing fatigue. *J Clin Virol* 37 Suppl 1:S33-8
Vermeulen RC, Scholte HR. 2006. Azithromycin in chronic fatigue syndrome (CFS), an analysis of clinical data. *J Transl Med* 4:34
Gonzalez-Rey E, Delgado M. 2005. Role of vasoactive intestinal peptide in inflammation and autoimmunity. *Curr Opin Investig Drugs* 6:1116-23
Janas E, Priest R, Malhotra R. 2005. Functional role of lipid rafts in CD20 activity? *Biochem Soc Symp*:165-75
Glennie MJ, French RR, Cragg MS, Taylor RP. 2007. Mechanisms of killing by anti-CD20 monoclonal antibodies. *Mol Immunol* 44:3823-37
Coiffier B, Lepage E, Briere J, Herbrecht R, Tilly H, Bouabdallah R, et al. 2002. CHOP chemotherapy plus rituximab compared with CHOP alone in elderly patients with diffuse large-B-cell lymphoma. *N Engl J Med* 346:235-42
Pfreundschuh M, Trumper L, Osterborg A, Pettengell R, Trneny M, Imrie K, et al. 2006. CHOP-like chemotherapy plus rituximab versus CHOP-like chemotherapy alone in young patients with good-prognosis diffuse large-B-cell lymphoma: a randomised controlled trial by the MabThera International Trial (MInT) Group. *Lancet Oncol* 7:379-91

Marcus R, Imrie K, Belch A, Cunningham D, Flores E, Catalano J, et al. 2005. CVP chemotherapy plus rituximab compared with CVP as first-line treatment for advanced follicular lymphoma. *Blood* 105:1417-23
van Oers MH, Klasa R, Marcus RE, Wolf M, Kimby E, Gascoyne RD, et al. 2006. Rituximab maintenance improves clinical outcome of relapsed/resistant follicular non-Hodgkin lymphoma in patients both with and without rituximab during induction: results of a prospective randomized phase 3 intergroup trial. *Blood* 108:3295-301
Dass S, Vital EM, Emery P. 2006. Rituximab: novel B-cell depletion therapy for the treatment of rheumatoid arthritis. *Expert Opin Pharmacother* 7:2559-70
Sanz I, Anolik JH, Looney RJ. 2007. B cell depletion therapy in autoimmune diseases. *Front Biosci* 12:2546-67
Dorner T, Lipsky PE. 2007. B-cell targeting: a novel approach to immune intervention today and tomorrow. *Expert Opin Biol Ther* 7:1287-99
Kavanaugh AF. 2006. B cell targeted therapies: safety considerations. *J Rheumatol Suppl* 77:18-23
Solal-Celigny P. 2006. Safety of rituximab maintenance therapy in follicular lymphomas. *Leuk Res* 30 Suppl 1:S16-21
Wagner SA, Mehta AC, Laber DA. 2007. Rituximab-induced interstitial lung disease. *Am J Hematol* 82:916-9
Edwards JC, Cambridge G, Leandro MJ. 2006. B cell depletion therapy in rheumatic disease. *Best Pract Res Clin Rheumatol* 20:915-28

Drug intervention in Chronic Fatigue Syndrome

Enquiry for participation in a study of drug testing in Chronic Fatigue Syndrome

Haukeland University Hospital, April 7th 2008

Background and aim

This is an enquiry for your possible participation in a scientific study testing the drug Rituximab (MabThera®) for Chronic Fatigue Syndrome (CFS)/ Myalgic encephalomyelitis (ME), which you have been diagnosed having through a previous medical examination. Since this disease today has no generally recognized and effective treatment, and there are no laboratory analyses that are used to disclose the diagnosis or follow the course of the treatment, it is necessary to register continuously over time how the patients feel after treatment has been given to find out how the disease is affected by the drug. The reason for utilizing this drug is the assumption that an over-reactivity in the body's immune system is an important factor in this disease. In addition, a few pilot patients have been given Rituximab treatment with a seemingly positive effect.

The reason for asking you to participate is knowledge of your disease through previous investigations at the hospital. The drug testing is done at the Department of Oncology at Haukeland University Hospital, in collaboration with the Department of Neurology. The aim of the study is to find a treatment that can improve the symptoms associated with Chronic Fatigue Syndrome.

What does the study imply?

In the study a minimum of 30 patients with Chronic Fatigue Syndrome will be electronically drawn (randomised) to receive Rituximab or an inactive agent (Placebo) with equal chances of getter either intervention. Since the symptoms the patients experience best measure the disease activity, it is important that neither the patient nor the physician know what treatment is given to each patient, in order to minimize the influence of expectations of an effect on the symptoms. Even though the study is performed without the patients or physician knowing which drug (Rituximab or Placebo) is given, this information on the drug can be attained if a medical reason for this arises.

After examination by the physician and blood tests, which aim at eliminating other conditions that exclude participation in the study, you will be randomised to either receive Rituximab or Placebo (saline water) two times with a two weeks interval, given as an intravenous infusion over a few hours at the Oncology ward. The treatment will be given under nurse surveillance. We request you to stay at the ward until the next day for observation.

The study necessitates registration of the symptom changes you experience every second week during the 12 months follow-up period, in accordance with the forms you have received. You will have to meet at the outpatient clinic for blood tests at 2 and 4 weeks after intervention, and then for consultations with the physician at 2, 3, 4, 6, 8, 10 and 12 months.

Since cognition of how the CFS disease is perceived may be influenced by psychological factors, we also wish to register through a questionnaire, in up to 20 patients included the study, the occurrence of depressive symptoms at inclusion and at 4 months follow-up. You may be asked verbally to participate in that part of the study.

Pregnancy excludes participation in the study and secure contraception must be used before start of and the first 12 months after the treatment. Breast-feeding women cannot participate.

It is advisable that no other medication that could influence Chronic Fatigue symptoms be used in the observation period of 4 months following inclusion. Drugs needed for other disease that occur during the study can be used if necessary, but these must not be instituted without conference with the responsible physicians. All medication used at start of the study and changes instituted during the study period will be registered.
If you do not wish to participate in the study, you will be offered follow-up according to usual clinical practice by your primary care physician in cooperation with the Department of Neurology.

Possible advantages, disadvantages and serious side effects
The advantage of participating in the study is that you may be offered a treatment that may prove to improve your well-being and give relief from the disease symptoms. You will also contribute to increased knowledge on handling of the disease and thereby help future patients. Rituximab is a well known drug used for treating lymphoma in recent years. It is a so-called monoclonal antibody directed toward white blood cells called B-lymphocytes. The most frequent side effects (seen in 10-15%) are reactions during the infusions, consisting of temporary skin rashes (urticarial, nettle-rash) with itching, and nausea, headache, hypotension and fever. Rarely breathing problems with need of intervention can arise. As when using other drugs, rare side effects that are not anticipated can arise. Your physician can inform you of this. Since this drug has not previously been used for chronic fatigue syndrome, we do not know for certain which side effects that could arise. A theoretical risk is activation of an ongoing infection when using Rituximab. For this reason, a number of blood tests are analysed before the start of treatment to diminish the risk of you having a concealed infection.

Privacy and research biobank
The tests drawn and the information registered about you will solely be used as described in the aims of this study. All information and tests in connection with research will be handled without name and personal identity number or other directly identifying information. A code connects you to the information and tests through a list of names. This list that connects your name to the code will be kept at the Department of Oncology and only persons involved in the study will have access to it. When the results of the study are published in a scientific journal, it will not be possible to disclose your identity. The data will be extinguished 15 years after the final results of the study are published.
If you during the course of the study wish to withdraw from the study, the information about you up to that point will be a part of the study material. Blood tests taken in the study and the information derived from that material will be stored in a research biobank, and it is a condition that you by participating in the study also give your consent to inclusion of the biological material and results derived thereof in the biobank. The study chairman Olav Mella is responsible for the biobank. Material from this study will among other analyses also be investigated on genetic changes in white blood cells in relation to Chronic Fatigue Syndrome. Aims of these genetic analyses are to find the mechanisms behind the disease by registering which genes that are activated when the disease is symptomatic and how these change if the treatment influences symptoms. These analyses will initially not have consequences for individual patients, but hopefully they will contribute to finding even better treatment for patients with Chronic Fatigue Syndrome in the future.

We draw your attention to the fact that governmental authorities may check information given in the study and see if it matches information from your patient files. This is to control the quality of the study. All information about you will be handled confidentially.

Right of inspection and keeping of the material
If you agree to participate in the study, you are granted the right of inspection into which information that is registered about you. You will have the right to change erroneous information that might have been gathered and registered. If you withdraw from the study, no more information or material will be gathered about you. The information already gathered will not be obliterated.

Financing
The study and biobank are financed by Helse Bergen and research money in the Department of Oncology, without any support from the pharmaceutical industry.

Insurance
You are insured according to the Product Liability Act in the Liability Insurance in connection with clinical trials with drugs.

Information on the trial results
You have the right to be informed on the outcome of the study. At earliest 4 months after the final patient has been included in the study, the trial leadership may open the code on which treatment that has been given and you will be informed on which treatment you have received as soon as it is practical to do so. When a final report is available from the study, you will be informed on the outcome of the study as a whole. If the study proves that Rituximab had a beneficial (significant) effect on the symptoms patients had at study inclusion, you will, if it proves you were given Placebo (inactive drug), be offered the active drug, but then in a new study that will be different from the present.

Voluntary participation
It is voluntary to participate in the study. You can at any time and without having to give a reason withdraw from the study, without that having consequences for your further follow-up. You will sign a consent formula if you decide to participate in the study.

Additional information on the study
If you have questions concerning the study, take contact with the Department of Oncology (55 97 20 10) and ask for Consultant Øystein Fluge or Professor Olav Mella, or for the Department of Neurology (telephone 55 97 50 44) and ask for Consultant Halvor Næss, Consultant Annette Storstein or Professor Harald Nyland.

Consent of participation in the study
I am willing to participate in the study

…………………………………………………………………………………………………
(signed by the participant, date)

Verification that the information has been given to the participant
I verify having verbally informed about the study

…………………………………………………………………………………………………...
(signed, role in study, date)

Baseline selfreported symptom registration (the last three months)

Scale 1-10: 1; no symptom…**5;** moderate symptom,… **10;** extreme symptom

	**Before intervention**
**Date**	
**FATIGUE**	
Fatigue	
Post-exertional exhaustion	
Need for rest	
Lack of daily functioning	
**PAIN**	
Muscle pain	
Headache	
Joint pain	
Cutaneous pain	
**COGNITIVE**	
Memory problems	
Ability to concentrate	
Mental tiredness	
Mood instability	
Ability to read	
Ability to watch television or data	
**OTHER SYMPTOMS**	
Sleep disturbances	
Nausea	
Diarrhoea	
Constipation	
Urinary bladder dysfunction	
Dizziness	
Intolerance to light	
Intolerance to noise	
Visual disturbances	
Sweating	
Palpitations	
Mouth dryness	
Rash	
Tender lymph nodes	
Temperature regulation disturbance	
HOW HAS THE DISEASE AFFECTED YOUR QUALITY OF LIFE THE LAST 3 MONTHS	
HOW DO YOU INTERPRETE THE ”OVERALL CFS” STATUS THE LAST 3 MONTHS	

Selfreported symptom change, every second week

__Scale__: **0**: major worsening, **1**: moderate worsening, **2**: slight worsening, __3: no change__, **4**: slight improvement, **5**: moderate improvement, **6**: major improvement

(continued until 52 weeks)

**Weeks from intervention**	**0**	**2**	**4**	**6**	**8**	**10**	**12**	**14**	**16**
**Date**									
**FATIGUE**									
Fatigue	3								
Post-exertional exhaustion	3								
Need for rest	3								
Lack of daily functioning	3								
**PAIN**									
Muscle pain	3								
Headache	3								
Joint pain	3								
Cutaneous pain	3								
**COGNITIVE**									
Memory problems	3								
Ability to concentrate	3								
Mental tiredness	3								
Mood instability	3								
Ability to read	3								
Ability to watch television or data	3								
**OTHER SYMPTOMS**									
Sleep disturbances	3								
Nausea	3								
Diarrhoea	3								
Constipation	3								
Urinary bladder dysfunction	3								
Dizziness	3								
Intolerance to light	3								
Intolerance to noise	3								
Visual disturbances	3								
Sweating	3								
Palpitations	3								
Mouth dryness	3								
Rash	3								
Tender lymph nodes	3								
Temperature regulation disturbance	3								
HOW HAS THE DISEASE AFFECTED YOUR QUALITY OF LIFE THE LAST 2 WEEKS	3								
HOW DO YOU INTERPRETE THE ”OVERALL CFS” STATUS THE LAST 2 WEEKS	3								

SF-36 QUESTIONNAIRE

(1992 -- Medical Outcomes Trust)

Patient Name: ______________________________ **Date: _____________________**

1. In general, would you say your health is: (circle one)

   Excellent Very good Good Fair Poor

2. <u>Compared to one year ago</u>, how would you rate your health in general <u>now</u>? (circle one)

   Much better now than one year ago.

   Somewhat better now than one year ago.

   About the same as one year ago.

   Somewhat worse than one year ago.

   Much worse than one year ago.

3. The following items are about activities you might do during a typical day. Does your health now limit you in these activities? If so, how much? (Mark each answer with an **X**)

**<u>ACTIVITIES</u>**	**Yes, Limited A Lot**	**Yes, Limited A Little**	**No, Not Limited At All**
a. **Vigorous activities,** such as running, lifting heavy objects, participating in strenuous sports			
b. **Moderate activities,** such as moving a table, pushing a vacuum cleaner, bowling, or playing golf			
c. Lifting or carrying groceries			
d. Climbing **several** flights of stairs			
e. Climbing **one** flight of stairs			
f. Bending, kneeling or stooping			
g. Walking **more than a mile**			
h. Walking **several blocks**			
i. Walking **one block**			
j. Bathing or dressing yourself			

4. During the past 4 weeks, have you had any of the following problems with your work or other regular daily activities as a result of your physical health? (Mark each answer with an **X**)

	**YES**	**NO**
a. Cut down on the **amount of time** you spent on work or other activites		
b. **Accomplished less** than you would like		
c. Were limited in the **kind** of work or other activities		
d. Had **difficulty** performing the work or other activities (for example, it took extra effort)		

5. During the past 4 weeks, have you had any of the following problems with your work or other regular daily activities as a result of any emotional problems (such as feeling depressed or anxious)? (Mark each answer with an **X**)

	**YES**	**NO**
a. Cut down the **amount of time** you spent on work or other activities		
b. **Accomplished less** than you would like		
c. Didn't do work or other activities as **carefully** as usual		

6. During the past 4 weeks, to what extent has your physical health or emotional problems interfered with your normal social activities with family, friends, neighbors or groups? (circle one)

Not at all Slightly Moderately Quite a bit Extremely

7. How much bodily pain have you had during the past 4 weeks? (circle one)

None Very mild Mild Moderate Severe Very severe

8. During the past 4 weeks, how much did pain interfere with your normal work (including both work outside the home and housework)?

Not at all A little bit Moderately Quite a bit Extremely

9. These questions are about how you feel and how things have been with you during the <u>past 4 weeks</u>. For each question, please give the one answer that comes closest to the way you have been feeling. How much of the time during the <u>past 4 weeks</u> – (Mark each answer with an **X**)

	**All of the Time**	**Most of the Time**	**A Good Bit of the Time**	**Some of the Time**	**A Little of the Time**	**None of the Time**
a. Did you feel full of pep?						
b. Have you been a very nervous person?						
c. Have you felt so down in the dumps that nothing could cheer you up?						
d. Have you felt calm and peaceful?						
e. Did you have a lot of energy?						
f. Have you felt downhearted and blue?						
g. Did you feel worn out?						
h. Have you been a happy person?						
i. Did you feel tired?						

10. During the <u>past 4 weeks</u>, how much of the time has your <u>physical health or emotional problems</u> interfered with your social activities (like visiting with friends, relatives, etc.)? (circle one)

All of the time   Most of the time   Some of the time   A little of the time   None of the time

11. How TRUE or FALSE is <u>each</u> of the following statements for you?

	**Definitely True**	**Mostly True**	**Don't Know**	**Mostly False**	**Definitely False**
a. I seem to get sick a little easier than other people					
b. I am as healthy as anybody I know					
c. I expect my health to get worse					
d. My health is excellent					

Physician assessed CFS symptoms at baseline (scale 1-10), and symptom change during follow-up (scale 0-6)

Scale	*1 - 10*	*1 - 10*		*0-6*	*0 - 6*	*0-6*	*0 - 6*	*0-6*	*0-6*	*0 - 6*
	**Baseline Oncol**	**Baseline Neurol**		**2 months Oncol**	**3 months Oncol**	**4 months Neurol**	**6 months Oncol**	**8 months Oncol**	**10 months Oncol**	**12 months Oncol**
**Date**										
**FATIGUE**										
Fatigue										
Post-exertional exhaustion										
Need for rest										
Lack of daily functioning										
**PAIN**										
Muscle pain										
Headache										
Joint pain										
**COGNITIVE**										
Memory problems										
Ability to concentrate										
Mental tiredness										
Mood instability										
**OTHER**										
Sleep disturbances										
Dizziness										
Temperature regulation disturbance										
Diarrhoea										

Baseline: **scale 1-10** **(see previous page)**

Follow-up (2, 3, 4, 6, 8, 10, 12 months)
Scale: **0**; major worsening, **1**: moderate worsening, **2**; slight worsening, 3; no change, **4**; slight improvement, **5**; moderate omprovement; **6**: major improvement

Rituximab

Kronisk tretthetssyndrom. Studie.

Medikament	Dose	CFS
Rituximab	500 mg/m^2 iv<br>(max 1000 mg)	Dag 1 og dag 15

- Rituximab blandes i NaCl 9 mg/ml slik at blandingen får konsentrasjon 2 mg/ml.

- Ferdig tilberedt infusjonsløsning av Rituximab bør brukes umiddelbart.
  Den er stabil i 12 timer ved romtemperatur. Om nødvendig kan ferdig oppløsning oppbevares i 24 timer i kjøleskap.

- *Behandling nr 1:*
  Initial infusjonshastighet er 50 mg/t i 1 time, deretter kan infusjonshastigheten økes med 50 mg/t hvert 30 minutt til maksimalt 400 mg/t.

- *Behandling nr 2 og senere:*
  Initial infusjonshastighet er 100 mg/t i 1 time, deretter kan infusjonshastigheten økes med 100 mg/t hvert 30 minutt til maksimalt 400 mg/t.

- Premedikasjon (1 time før Rituximab infusjonen starter):
  Paracet® 1 g po
  Zyrtec® 10 mg po
  Decadron® 8 mg po

- Beredskap (skal være lett tilgjengelig på pasientrommet):
  Adrenalin®
  Solu-Cortef®
  Aminophyllin®
  Dexclorpheniramin®
  Nødvendige sprøyter / sprøytespisser
  1000 ml NaCl 0,9% m/iv-sett

- Temperatur taes hvert 30. minutt og ellers ved kliniske symptomer som skulle tilsi feberutvikling.

- BT og puls taes hvert 15. minutt de første to timene. I denne perioden må sykepleier være kontinuerlig tilstede hos pasienten.

  Hvis pasienten er klinisk ustabil etter 2 timer, taes BT og puls fortsatt hvert 15. minutt - inntil pasienten er klinisk stabil. Sykepleier må fortsatt være kontinuerlig tilstede hos pasienten.

  Hvis pasienten er klinisk stabil etter 2 timer, kan BT og puls taes hvert 30. minutt videre. Sykepleier må hyppig tilse pasienten.

OBSERVASJONER VED RITUXIMAB-INFUSJON:

- Bivirkningene er i hovedtrekk relatert til «cytokine release syndrome» (hyppigst) og allergi. Cytokine release vil ikke være særlig relevant for CFS pasienter som ikke har lymfom-manifestasjoner. Symptomene på disse to tilstander kan være svært like!
- Ved utvikling av bivirkninger - kontakt lege!
  Bivirkninger finner først og fremst sted de første to timer av infusjonen.
  Det kan være aktuelt å avbryte infusjonen, redusere infusjonshastigheten eller f.eks gi tilleggsmedikasjon.

- Vær spesielt oppmerksom på følgende symtomer og funn:
  - Dyspnoe / bronchospasme / hypoxi
  - Feber / frysninger
  - Urticaria / angioødem (hevelse i hals / tunge)
  - Hypotensjon / arytmi

  Andre symptomer og funn:
  - Flushing, kvalme / oppkast, tretthet, hodepine, rhinitt / irritasjon i hals, smerte i tumor / infusjonsstedet

Infusjonsskjema for 1. Rituximab-infusjon:

Klokken	ml/t	mg/t	totalt infundert	infusjonstid	BT / puls / temp
	25 ml/t	50 mg/t	50 mg	60 min	
	50 ml/t	100 mg/t	100 mg	30 min	
	75 ml/t	150 mg/t	175 mg	30 min	
	100 ml/t	200 mg/t	275 mg	30 min	
	125 ml/t	250 mg/t	400 mg	30 min	
	150 ml/t	300 mg/t	550 mg	30 min	
	175 ml/t	350 mg/t	725 mg	30 min	
	200 ml/t	400 mg/t		videre	

KLINISK UTPRØVNING KTS-1-2008

Ansvarlig lege: Dr. Mella Produksjonsnr: ....

For dato: 09.04.08

Dato...................kl....................Sign..............Anv.senest..............

Holdbarhet: 12 t i rom.temp og 24 t kjølig etter utblanding.

Tilsatt legemiddel: Mengde:

Mabthera/Placebo xxx mg

Infusjonen påbegynt kl..................Avsluttet kl.....................

Avdeling: Kreft 2 vest **Pas. nr:**

Navn: **Infusjonsnr.:**

F.dato:

Haukeland sykehusapotek
Apotekene Vest
